# A Bayesian estimation method for variational phase-field fracture problems

**DOI:** 10.1007/s00466-020-01876-4

**Published:** 2020-07-14

**Authors:** Amirreza Khodadadian, Nima Noii, Maryam Parvizi, Mostafa Abbaszadeh, Thomas Wick, Clemens Heitzinger

**Affiliations:** 1 Institute of Analysis and Scientific Computing, Vienna University of Technology (TU Wien), Wiedner Hauptstraße 8–10, 1040 Vienna, Austria; 2 Faculty of Mathematics and Computer Sciences, Amirkabir University of Technology, No. 424, Hafez Ave., Tehran, 15914 Iran; 3 Institute of Applied Mathematics, Leibniz University Hannover, Welfengarten 1, 30167 Hanover, Germany; 4 School of Mathematical and Statistical Sciences, Arizona State University, Tempe, AZ 85287 USA

**Keywords:** Bayesian estimation, Inverse problem, Phase-field propagation, Brittle fracture, Multi-field problem

## Abstract

In this work, we propose a parameter estimation framework for fracture propagation problems. The fracture problem is described by a phase-field method. Parameter estimation is realized with a Bayesian approach. Here, the focus is on uncertainties arising in the solid material parameters and the critical energy release rate. A reference value (obtained on a sufficiently refined mesh) as the replacement of measurement data will be chosen, and their posterior distribution is obtained. Due to time- and mesh dependencies of the problem, the computational costs can be high. Using Bayesian inversion, we solve the problem on a relatively coarse mesh and fit the parameters. In several numerical examples our proposed framework is substantiated and the obtained load-displacement curves, that are usually the target functions, are matched with the reference values.

## Introduction

This work is devoted to parameter identifications in fracture failure problems. To formulate fracture phenomena, a phase-field formulation for quasi-brittle fracture is used. The variational phase-field formulation is a thermodynamically consistent framework to compute the fracture failure process. This formulation emanates from the regularized version of the sharp crack surface function, which was first modeled in a variational framework in [1]. Regularized fracture phenomena are described with an additional auxiliary smooth indicator function [2], which is denoted as crack phase-field (here indicated by *d*). Along with a mechanical field (denoted by $\textit{\textbf{u}}$), a minimization problem for the multi-field problem $(\textit{\textbf{u}},d)$ can be formulated. The main feature of such a variational formulation is to approximate the discontinuities in $\textit{\textbf{u}}$ across the lower-dimensional crack topology with the phase-field function *d*.

The resulting, regularized formulation leads to a diffusive transition zone between two phases in the solid, which corresponds to the fractured phase (i.e., $d=0$) and intact phase (i.e., $d=1$), respectively. The transition zone is determined by the phase-field regularization parameter $\ell$, also well-known as the length-scale parameter. The parameter $\ell$ is related to the element size *h* and specifically $h\le \ell$ (e.g., $\ell =2h$). Therefore, sufficiently small length-scales are computationally demanding. To date, the focus in such cases was on local mesh adaptivity and parallel computing in order to reduce the computational cost significantly; see for instance [3–11]. Another recent approach is a global-local technique in which parts of the domain are solved with a simplified approach [12, 13] that also aims to reduce the computational cost.

Generally, material parameters fluctuate randomly in space. In fact, the mechanical material parameters are spatially variable and, therefore, the uncertainty related to spatially varying properties can be represented by random fields. For instance, the material stiffness property has spatial variability. In fact, there are several sources of uncertainty including the class of extensometer or strain gauge resolution, uncertainty in the dimensional measurements, the classification and resolution of the load cell, misalignment of the specimen or strain measurement device, temperature effects, operator-dependent factors, data fitting routines and analysis methods, etc [14]. Therefore, in order to provide a reliable model, the uncertainty effect must be taken into account.

The main goal in this work is to identify such uncertain parameters for phase-field fracture problems. The underlying framework of parameter estimation using Bayesian inference is described in the following. Bayesian inference is a probabilistic method used to estimate the unknown parameters according to the prior knowledge. The observations (experimental or synthetic measurements) can be used to update the prior data and provide the posterior estimation. The distribution provides useful information about the possible range of parameters and their variations and mean. Markov chain Monte Carlo (MCMC) [15] is a common computational approach for extracting information of the inverse problem (posterior distribution). Metropolis-Hastings (MH) algorithm [16] is the most popular MCMC method to generate a Markov chain employing a proposal distribution for new steps. In practice, a reliable estimation of influential parameters is not possible or needs significant efforts. In [17, 18], the authors used the Metropolis-Hastings algorithm to estimate the unknown parameters in field-effect sensors. It enables authors to estimate probe-target density of the target molecules which can not be experimentally estimated. We refer interested readers to [19, 20] for more applications of Bayesian estimation. In the same line, other optimization approaches can be used to determine intrinsic material properties of the specimen from experimental load-displacement curves, see e.g., [21].

As previously mentioned, we consider fractures in elastic solids in this work. The principal material parameters are the shear modulus $\mu$ and the effective bulk modulus, $K=\lambda + \frac{2\mu }{3}$ (here $\lambda$ denotes Lamé’s first parameter) and Griffith’s critical energy release rate $G_c$. Using Bayesian inversion, the objective is to determine the unknown elasticity parameters.

For a homogeneous material, the stability requires positive-definiteness of the elasticity tensor. For an externally unconstrained homogeneous solid, the conditions of structural stability needs that the fourth-order stiffness tensor is positive-definite. The condition for an isotropic, linear elastic medium gives rise to the shear modulus $\mu$ and the effective bulk modulus, *K* be strictly positive [22]. Regarding $\lambda$, the bound $\lambda >-\frac{2\mu }{3}$ may relate it to the shear modulus. Also, for the isotropic materials (as used in this paper) Poisson’s ratio $\nu$ satisfies the condition $-1< \nu < \frac{1}{2}$ [23]. These two elasticity parameters ($\lambda$ and $\nu$) are not well-suited for the estimation due to their bounds and dependency. Therefore, for the elasticity parameterization, we chose the eigenvalues, i.e., *K* and $\mu$, and strive to estimate the joint probability, being updated jointly using MCMC.

**Fig. 1:**
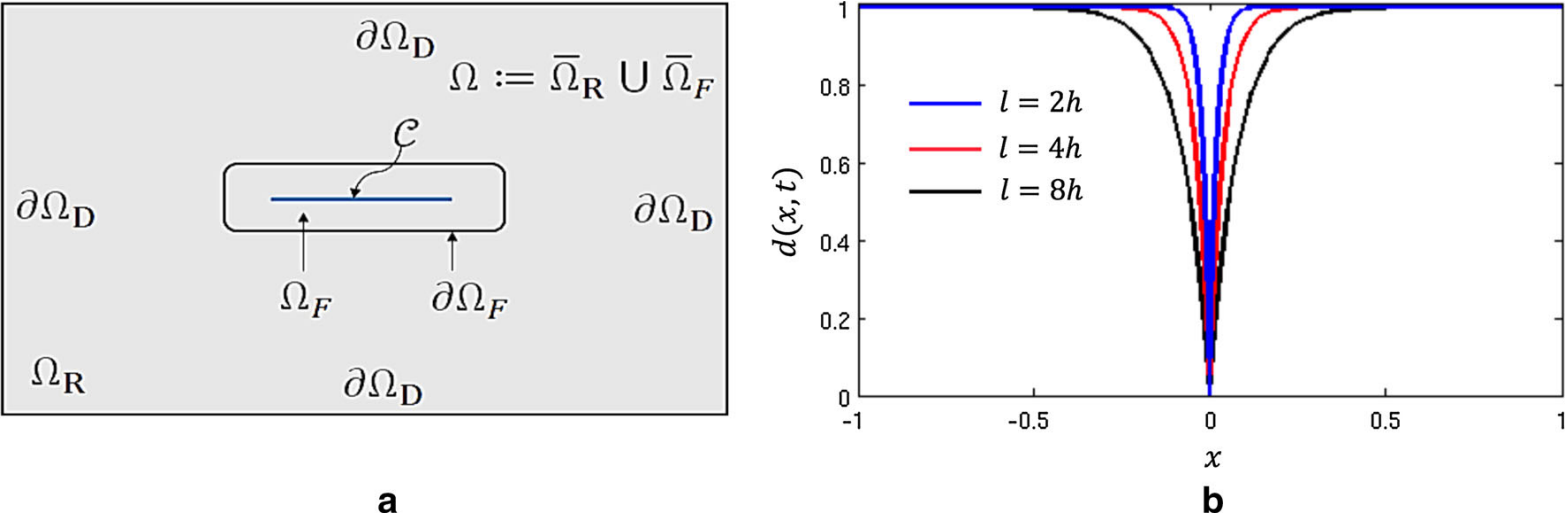
**a** Geometric setup: the intact region indicated by $\Omega _R$ and $\mathcal {C}$ is the crack phase-field surface. The entire domain is denoted by $\Omega$. The crack phase-field is approximated in the domain $\Omega _F$. The fracture boundary is $\partial \Omega _F$ and the outer boundary of the domain is $\partial \Omega$. $\Omega _F$ is represented by means of *d* such that the transition area is $0< d< 1$ with thickness $2\ell$. **b** Regularized crack phase-field profile for a different length scale. A smaller value for the length scale lets the crack phase-field profile converge to a delta distribution

Griffith’s theory describes that crack propagation occurs if a certain reduction of the potential energy due to the change of surface energy associated with incremental crack extension reaches to its critical value [24]. Here, Griffith’s critical energy rate $G_c$ measures the amount of energy dissipated in a localized fracture state [25], thus has units of energy-per-unit-area. In case $G_c$ is unknown, one possibility is to employ the Bayesian setting for its identification. Physically speaking, there is a direct relation between $G_c$ and material stiffness, which means that in stiffer materials more energy is needed for the crack initiation. Computationally speaking, this value is independent of the elasticity parameters. Finally, since we should deal with three positive values ($\mu$, *K*) and $G_c$, in order to remove the positivity constraints, we transfer these parameters and estimate the transfered values $\mu ^*=\log (\mu ), K^*=\log (K)$, and $G_c^*=\log (G_c)$.

In our Bayesian framework, a reference value (obtained on a sufficiently refined mesh which termed here to the $\textit{virtual observation}$) as the replacement of measurement will be chosen. Then, the posterior density of the elasticity parameters (joint probability) and the critical energy release rate is obtained. The computational costs can be high, specifically when an appropriate estimation is required inside multi-physics frameworks, see e.g. [3, 26–28]. Using Bayesian inversion, we strive to solve such problems with a coarser mesh and fit the parameters. The obtained load-displacement curve (as an important characteristic output) is matched with the reference value. In spite of using coarser meshes and therefore significantly lower computational costs (in terms of CPU timings), the accuracy of the solution is reliable (crack initiation and material fracture time estimated precisely).

The paper is organized as follows: In Sect. 2, we describe the variational isotropic phase-field formulation for the brittle fracture that is a thermodynamically consistent framework to compute the fracture failure process. In Sect. 3, the Bayesian inference is explained. We describe how the MH algorithm will be used to estimate the unknown parameters in phase-field fracture. Also we point out the critical points in the load-displacement curve, which must be estimated precisely with the Bayesian approach. In Sect. 4, the Bayesian framework is adopted to estimate unknown parameters in the phase-field fracture approach. In Sect. 5, three specific numerical examples with different parameters and geometry will be given. We will use two proposal distributions (uniform and normal distribution) to sample the candidates and estimate the unknown parameters with different mesh sizes. Finally, in 6 we will draw paper conclusions and explain our future planes for employing Bayesian inversion in heterogeneous materials.

## Variational isotropic phase-field brittle fracture

### The primary fields for the variational phase-field formulation

We consider a smooth, open and bounded domain $\Omega \subset \mathbb {R}^\delta ~(\text {here}~ \delta =2)$. In this computational domain, a lower dimensional fracture can be indicated by $\mathcal {C}\subset \mathbb {R}^{\delta -1}$. In the following, Dirichlet boundaries conditions indicated as $\partial \Omega _D := \partial \Omega$, and Neumann boundaries conditions are given on $\partial _N \Omega := \Gamma _N \cup \partial \mathcal {C}$, where $\Gamma _N$ is the outer boundary of $\Omega$ and $\partial \mathcal {C}$ is the crack boundary. The geometric setup including notations is illustrated in Fig. 1a. The surface fracture $\mathcal {C}$ is estimated in $\Omega _F\subset \Omega \subset \mathbb {R}^\delta$. A region without any fracture (i.e., an intact region) is indicated by $\Omega _R:=\Omega \backslash \Omega _F\subset \Omega \subset \mathbb {R}^\delta$ such that $\Omega _R\cup \Omega _F=\Omega$ and $\Omega _R\cap \Omega _F=\varnothing$.

The variational phase-field formulation is a thermodynamically consistent framework to compute the fracture process. Due to the presence of the crack surface, we formulate the fracture problem as a two-field problem including the displacement field $\textit{\textbf{u}}(\textit{\textbf{x}}):\Omega \rightarrow \mathbb {R}^\delta$ and the crack phase-field $d(\textit{\textbf{x}}):\Omega \rightarrow [0,1]$. The crack phase-field function $d(\textit{\textbf{x}})$ interpolates between $d=1$, which indicates undamaged material, and $d=0$, which indicates a fully broken material phase.

For stating the variational formulations, the spaces

1 $$\begin{aligned} \textit{\textbf{V}}:=&~\{ \mathbf{H}^1(\Omega )^\delta :\textit{\textbf{u}}=\bar{\textit{\textbf{u}}}\; \mathrm {on} \; \partial \Omega _D \}, \end{aligned}$$

2 $$\begin{aligned} W:=&~\text {H}^1(\Omega ) , \end{aligned}$$

3 $$\begin{aligned} W_{\mathrm{in}} :=&~ \{ d \in \text {H}^1(\Omega )^{\delta -1} : 0 \le d\le d^{\mathrm{old}} \} \end{aligned}$$

are used. Herein, $W_{\mathrm{in}}$ denotes a closed, non-empty and convex set which is a subset of the linear function space $W=\text {H}^1(\Omega )$ (see e.g., [29]).

### Variational formulation for the isotropic mechanical contribution

In the following, a variational setting for quasi-brittle fracture in bulk materials with small deformations is formulated. To formulate the bulk free energy stored in the material, we define the first and second invariants as

4 $$\begin{aligned} I_1(\varvec{\varepsilon })=tr(\varvec{\varepsilon }), \qquad I_2(\varvec{\varepsilon })=tr(\varvec{\varepsilon }^2), \end{aligned}$$

with the second-order infinitesimal small strain tensor defined as

5 $$\begin{aligned} \varvec{{\varepsilon }} = \nabla _s \textit{\textbf{u}} = \mathrm{sym}[ \nabla \textit{\textbf{u}} ]. \end{aligned}$$

The isotropic scalar valued free-energy function reads

6 $$\begin{aligned} {\widetilde{\Psi }}\left( I_1(\varvec{\varepsilon }),I_2(\varvec{\varepsilon })\right):= & {} \left( \frac{K}{2}\right) I^2_1(\varvec{\varepsilon }) -\mu ~\left( \frac{I^2_1(\varvec{\varepsilon })}{3}\right. \nonumber \\&\left. - I_2(\varvec{\varepsilon })\right) \quad \text {with}\quad K>0\quad \text {and}\nonumber \\&\mu >0,\nonumber \\ \end{aligned}$$

where $K= \lambda +\frac{2}{3}\mu$ is the bulk modulus. A stress-free condition for the bulk energy-density function requires ${\widetilde{\Psi }}\left( I_1(\textit{\textbf{0}}),I_2(\textit{\textbf{0}})\right) =0$. Hence, the bulk free-energy functional including the stored internal energy and the imposed external energy is

7 $$\begin{aligned} \begin{aligned} \mathcal {E}_{bulk}(\textit{\textbf{u}})=\int _{\Omega _C}{\widetilde{\Psi }}(\varvec{\varepsilon }) \mathrm {d}{\textit{\textbf{x}}}-\int _{{\partial _N\Omega _C }} {{\varvec{\tau }}} \cdot \textit{\textbf{u}}\,\mathrm {d}s \end{aligned} \end{aligned}$$

where $\varvec{\tau }$ is the imposed traction traction vector on $\partial _N\Omega _C := \Gamma _N \cup \mathcal {C}$ and the body-force is neglected.

Following [1], we define the total energetic functional which includes the stored bulk-energy functional and fracture dissipation as

8 $$\begin{aligned} \mathcal {E}(\textit{\textbf{u}},\mathcal {C})= \mathcal {E}_{bulk}(\textit{\textbf{u}}) + G_c \mathcal {H}^{\delta -1} (\mathcal {C}), \end{aligned}$$

where $G_c$ is the so called the Griffith’s critical elastic-energy release rate. Also, $\mathcal {H}^{\delta -1}$ refers to the $(\delta -1)$-dimensional Hausdorff measure (see e.g. [2]). Following [2], $\mathcal {H}^{\delta -1}$ is regularized (i.e. approximated) by the crack phase-field $d(\textit{\textbf{x}})$ (see e.g. [2]). Doing so, a second-order variational phase-field formulation is employed; see Sect. 2.3. Additional to that, a second-order stress degradation state function (*intacted-fractured transition* formulation) is used as a monotonically decreasing function which is lower semi-continuous order; see Sect. 2.5.

**Fig. 2:**
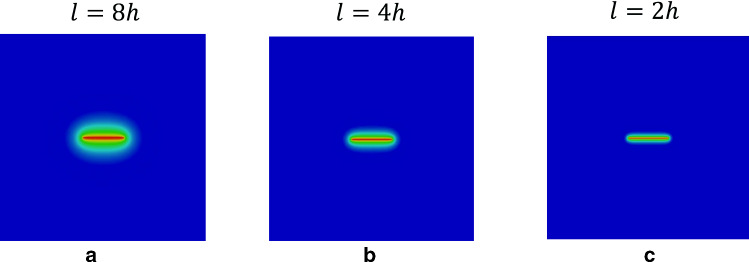
Effect of different length scales on the crack phase-field resolution as calculated by the minimization problem in Eq. 10 such that $\ell _a>\ell _b>\ell _c$

### Crack phase-field formulation in a regularized setting

Let us represent a regularized (i.e., approximated) crack surface for the sharp-crack topology (which is a Kronecker delta function) thorough the exponential function $d(\textit{\textbf{x}})=1-\exp ^{ - \vert \textit{\textbf{x}} \vert /l }$, which satisfies $d(\textit{\textbf{x}})=0$ at $\textit{\textbf{x}}=0$ as a Dirichlet boundary condition and $d(\textit{\textbf{x}})=1$ as $\textit{\textbf{x}}\rightarrow \pm \infty$. This is explicitly shown in Fig. 1b for different length scales. Here, $\textit{\textbf{x}}$ is a position variable in the Cartesian coordinate system, meaning $\textit{\textbf{u}}$ and *d* have a certain value at each position within the geometry. The first observation through the explicit formulation is that, the crack phase-field *d* constituting a smooth transition zone dependent on the regularization parameter $\ell$. In engineering or physics, $\ell$ is often a so-called characteristic length-scale parameter. This may be justified since this zone weakens the material and is a physical transition zone from the unbroken material to a fully damaged state. In practice, choices such as $\ell =2h$ or $\ell =4h$ are often employed. Following [30, 31], a regularized *crack surface energy functional* for the second term in Eq. 8 reads

9 $$\begin{aligned} G_c \mathcal {H}^{\delta -1} (\mathcal {C}):= & {} G_c\int _\Omega \gamma _\ell (d, \nabla d) \,\mathrm {d}{\textit{\textbf{x}}} \quad \text {with}\quad \gamma _{\ell }(d,\nabla d)\nonumber \\:= & {} \frac{\ell }{2\ell } {(1-d)^2} + \frac{\ell }{2} \nabla d \cdot \nabla d \end{aligned}$$

based on the crack surface density function $\gamma _{\ell }(d,\nabla d)$ per unit volume of the solid. This equation is the so-called AT-2 model because of the quadratic term in PDE.

We set sharp crack surfaces as Dirichlet boundary conditions in $\mathcal {C}\subset \Omega$. Hence, the crack phase-field $d(\textit{\textbf{x}},t)$ is obtained from the minimization of the regularized crack density function as

10 $$\begin{aligned} d(\textit{\textbf{x}})=\underset{d(\textit{\textbf{x}})\in W_{in} \; \text {with} \; d(\textit{\textbf{x}})=0 \; \forall \textit{\textbf{x}}\in \mathcal {C}}{\mathrm {argmin}} \int _\Omega \gamma _l(d, \nabla d) \,\mathrm {d}{\textit{\textbf{x}}}. \end{aligned}$$

Figure 2 gives the numerical solution that arises from the minimization Eq. 10 and demonstrates the effect of different regularized length scales on the numerical solution. Clearly, a smaller length scale leads to a narrower transition zone (see Fig. 2c). That is also in agreement with the crack phase-field profile shown in Fig. 1b.

### Strain-energy decomposition for the bulk free-energy

Fracture mechanics is the process which results in the compression free state. As a result, a fracture process behaves differently in the *positive phase* and in *negative phase*, see e.g. [32]. In the following, an additive split for the strain energy density function to distinguish the positive and negative phases is used. Instead of dealing with a full linearized strain tensor $\varvec{\varepsilon }(\textit{\textbf{u}})$, the additive decomposition

$$\begin{aligned} \varvec{\varepsilon }(\textit{\textbf{u}})=\varvec{\varepsilon }^{+}(\textit{\textbf{u}})+\varvec{\varepsilon }^{-}(\textit{\textbf{u}}) \quad \text {with}\quad \varvec{\varepsilon }^{\pm }(\textit{\textbf{u}}):=\sum _{i=1}^{\delta } \langle \varepsilon _i\rangle ^{\pm } \mathbf{N _i} \otimes \mathbf{N _i} \end{aligned}$$

of the strain tensor based on its eigenvalues is used [5, 31]. Herein, $\langle x \rangle _{\pm } := \frac{ x {\pm } |x|}{2}$ refers to the a Macaulay brackets for $x \in \mathbb {R}^\pm$. Furthermore, $\varvec{\varepsilon }^{+}$ and $\varvec{\varepsilon }^{-}$ refer to the positive and negative parts of the strain, respectively. The $\{\varepsilon _i\}$ are the principal strains (i.e., the eigenvalues of the $\varvec{\varepsilon }(\textit{\textbf{u}})$) and the $\{\mathbf{N }_i\}$ are the principal strain directions (i.e., the eigenvectors of the $\varvec{\varepsilon }(\textit{\textbf{u}})$). To determine the positive and negative parts of total strain ${\varvec{\varepsilon }}$, a positive-negative fourth-order projection tensor is

11 $$\begin{aligned} \mathbb {P}^\pm _{\varvec{\varepsilon }}:=\frac{\partial \varvec{\varepsilon }^\pm }{\partial \varvec{\varepsilon }}=\frac{\partial \left( \displaystyle \sum \nolimits _{i=1}^{\delta } \langle \varepsilon _i\rangle ^{\pm } \mathbf{N _i} \otimes \mathbf{N _i}\right) }{\partial \varvec{\varepsilon }}, \end{aligned}$$

such that the fourth-order projection tensor $\mathbb {P}^\pm _{\varvec{\varepsilon }}$ projects the total linearized strain ${\varvec{\varepsilon }}$ onto its positive-negative counterparts, i.e., $\varvec{\varepsilon }^{\pm }=\mathbb {P}^\pm _{\varvec{\varepsilon }}:\varvec{\varepsilon }$. Hence an additive formulation of the strain-energy density function consisting of the positive and the negative parts reads

12 $$\begin{aligned} {\Psi }\big (I_1(\varvec{\varepsilon }),I_2(\varvec{\varepsilon })\big ):= & {} \underbrace{{\widetilde{\Psi }}^{+}\big (I^{+}_1(\varvec{\varepsilon }),I^{+}_2(\varvec{\varepsilon })\big )}_{\text {tension term}}\nonumber \\&+\underbrace{{\widetilde{\Psi }}^{-}\big (I^{-}_1(\varvec{\varepsilon }),I^{-}_2(\varvec{\varepsilon })\big )}_{\text {compression term}}. \end{aligned}$$

Here, the scalar valued principal invariants in the positive and negative modes are

13 $$\begin{aligned} I_1^{\pm }(\varvec{\varepsilon }):=\langle {I_1(\varvec{\varepsilon })}\rangle _{\pm }, \quad I^{\pm }_2(\varvec{\varepsilon }):=I_2(\varvec{\varepsilon }^{\pm }). \end{aligned}$$

Here, the first positive/negative invariant $I_1(\varvec{\varepsilon })$ is strictly related to the tension/compression mode, respectively, meaning that if $\mathrm{tr}(\varvec{\varepsilon })>0$ requires that we are in tension mode otherwise we are in compression state. The second invariant $I_2(\varvec{\varepsilon })$ is mainly due to the positive and negative eigenvalue of the strain tensor, where its positive value requires that we are either in shear or in tension mode otherwise it is in compression. Thus, we distinguished between tension/compression and also a isochoric mode of our constitutive model, and only the positive part of the energy is degraded.

### Energy functional for the isotropic crack topology

Due to the physical response of the fracture process, it is assumed that the degradation of the bulk material due to the crack propagation depends only on the tensile and isochoric counterpart of the stored bulk energy density function. Thus, there is no degradation of the bulk material in negative mode, see [31]. Hence, the degradation function denoted as $g(d_+)$ acts only on the positive part of bulk energy given in Eq. 12, i.e.,

14 $$\begin{aligned} g(d_+):=d_+^2 ,\qquad g:[0,1]\rightarrow [0,1]. \end{aligned}$$

This function results in degradation of the solid during the evolving crack phase-field parameter *d*. Due to the transition between the intact region and the fractured phase, the degradation function has flowing properties, i.e.,

15 $$\begin{aligned}&g(0)=0,\quad g(1)=1,\quad g(d)>0 \;\; \text {for} \;\;d>0, \nonumber \\&\quad g'(0)=0,\quad g'(1)>0. \end{aligned}$$

Following [31], the small residual scalar $0<\kappa \ll 1$ is introduced to prevent numerical instabilities. It is imposed on the degradation function, which now reads

16 $$\begin{aligned} g(d_+):=(1-\kappa )d_+^2 + \kappa ,\qquad g:[0,1]\rightarrow [0,1). \end{aligned}$$

The stored bulk density function is denoted as $w_{bulk}$. Together with the fracture density function $w_{frac}$, it gives the the total density function

17 $$\begin{aligned} w(\varvec{\varepsilon }, d, \nabla d) = w_{\mathrm{bulk}} (\varvec{\varepsilon }, d) + w_{\mathrm{frac}} (d, \nabla d), \end{aligned}$$

with

18 $$\begin{aligned}&\quad w_{\mathrm{bulk}} (\varvec{\varepsilon }, d)=g(d_+){\widetilde{\Psi }}^{+}\big (I^{+}_1(\varvec{\varepsilon }),I^{+}_2(\varvec{\varepsilon })\big )\nonumber \\&\qquad \qquad \qquad \quad +{\widetilde{\Psi }}^{-}\big (I^{-}_1(\varvec{\varepsilon }),I^{-}_2(\varvec{\varepsilon })\big ),\nonumber \\&w_{\mathrm{frac}}(d, \nabla d) = G_c \gamma _l(d, \nabla d). \end{aligned}$$

#### Formulation 2.1

(Energy functional for isotropic crack topology) We assume that *K* and $\mu$ are given as well as initial conditions $\textit{\textbf{u}}_0=\textit{\textbf{u}}(\textit{\textbf{x}},0)$ and $d_0=d(\textit{\textbf{x}},0)$. For the loading increments $n \in \{1,2,\ldots , N\}$, find $\textit{\textbf{u}}:=\textit{\textbf{u}}^n\in V$ and $d:=d^n\in W_{in}$ such that the functional

$$\begin{aligned} \mathcal {E} ( {\textit{\textbf{u}}},d)&=\mathcal {E}_{\mathrm{bulk}}(\textit{\textbf{u}},d_+,\chi )+\; \mathcal {E}_{\mathrm{frac}}(d)+\;\mathcal {E}_{\mathrm{ext}}(\textit{\textbf{u}})\\&=\underbrace{\int _\Omega g(d_+)\;{\widetilde{\Psi }}^{+}(I^{+}_1,I^{+}_2) +{\widetilde{\Psi }}^{-}(I^{-}_1,I^{-}_2)\; \mathrm {d}{\textit{\textbf{x}}}}_{\text {bulk term}} \\&\quad + G_c\underbrace{\int _\Omega \gamma _{l}(d, \nabla d)\mathrm {d}{\textit{\textbf{x}}}}_{\text {fracture term}} -\underbrace{\int _{\partial _N\Omega } {\varvec{{\bar{\tau }}}} \cdot \textit{\textbf{u}}\,\mathrm {d}s}_{\text {external load}}, \end{aligned}$$

is minimized.

Herein, to make sure that phase-field quantity *d* lies in the interval [0, 1], we define $d_+$ to map negative values of *d* to positive values. In Formulation 2.1, the stationary points of the energy functional are determined by the first-order necessary conditions, namely the Euler–Lagrange equations, which can be found by differentiation with respect to ${\textit{\textbf{u}}}$ and *d*.

#### Formulation 2.2

(Euler–Lagrange equations) Let $K>0$, $\mu >0$ be given as well as the initial conditions $\textit{\textbf{u}}_0=\textit{\textbf{u}}(\textit{\textbf{x}},0)$ and $d_0=d(\textit{\textbf{x}},0)$. For the loading increments $n \in \{1,2,\ldots , N\}$, find $\textit{\textbf{u}}:=\textit{\textbf{u}}^n\in V$ and $d:=d^n\in W_{in}$ such that

19 $$\begin{aligned} \begin{aligned}&{{\mathcal {E}}}_{\textit{\textbf{u}}}(\textit{\textbf{u}}, d;\delta \textit{\textbf{u}})= \int _\Omega g(d_+) {\varvec{{\widetilde{\sigma }}}^{\mathrm{iso},+}}(\textit{\textbf{u}}): {\varvec{\varepsilon }}(\delta \textit{\textbf{u}})\mathrm {d}{\textit{\textbf{x}}}\\&\quad + \int _\Omega {\varvec{{\widetilde{\sigma }}}^{\mathrm{iso},-}(\textit{\textbf{u}})}: {\varvec{\varepsilon }}(\delta \textit{\textbf{u}})\mathrm {d}{\textit{\textbf{x}}}\\&\quad -\int _{\partial _N\Omega } {\varvec{{\bar{\tau }}}} \cdot {\delta \textit{\textbf{u}}}\,\mathrm {d}s= 0 \qquad \forall {\delta \textit{\textbf{u}}}\in V, \\&{{\mathcal {E}}}_d(\textit{\textbf{u}},d;\delta d-d) =(1-\kappa )\int _\Omega 2d_+ {\widetilde{D}}. (\delta d-d)\mathrm {d} {\textit{\textbf{x}}}\\&\quad + G_c \int _\Omega \left( \frac{1}{\ell }(d-1)\cdot (\delta d-d) + \ell \nabla d\cdot \nabla (\delta d-d)\right) \\&\quad \mathrm {d}{\textit{\textbf{x}}}\ge 0 \qquad \forall \delta d\in W \cap L^{\infty }. \end{aligned} \end{aligned}$$

Herein, ${{\mathcal {E}}}_{\textit{\textbf{u}}}$ and ${{\mathcal {E}}}_d$ are the first directional derivatives of the energy functional ${{\mathcal {E}}}$ given in Formulation 2.1 with respect to the two fields, i.e., $\textit{\textbf{u}}$ and *d*, respectively. Also, ${\widetilde{D}}$ is a crack driving state function which depends on a state array of strain- or stress like quantities and $\delta \textit{\textbf{u}}\in \{ \mathbf{H}^1(\Omega )^2: \delta \textit{\textbf{u}}=\textit{\textbf{0}} \; \mathrm {on} \; \partial \Omega _D \}$ is the deformation test function and $\delta d\in H^1(\Omega )$ is the phase-field test function.

**Fig. 3:**
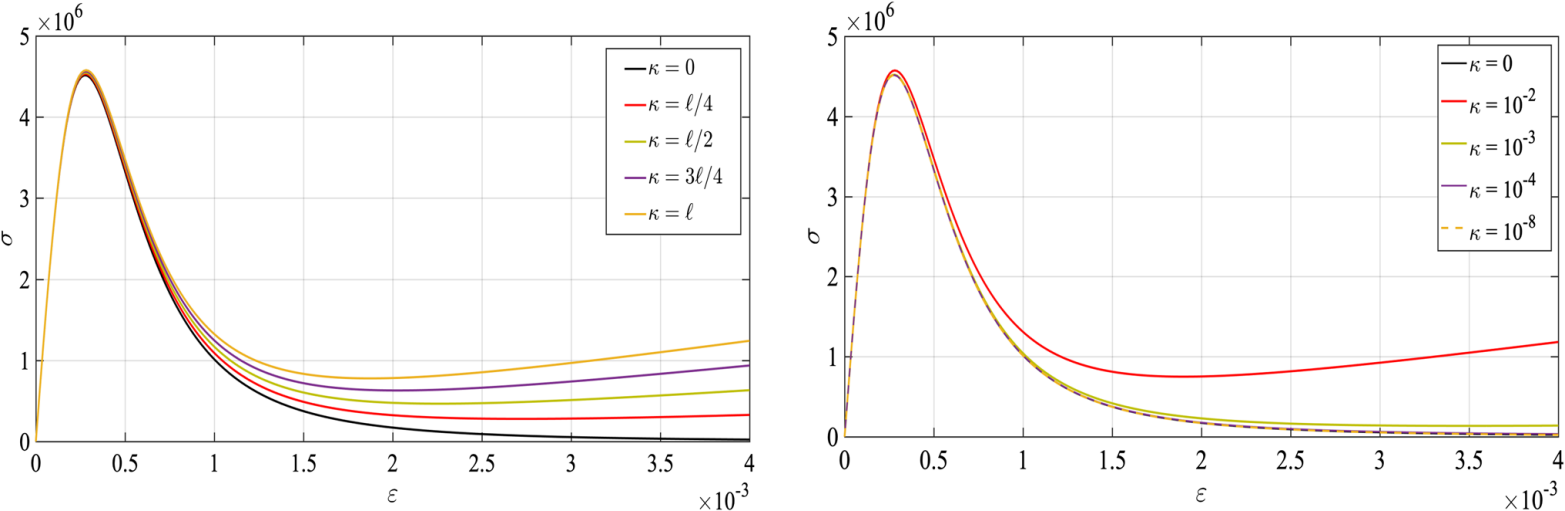
The influence of the $\kappa$ on the stress-strain curve; left plot represent $\kappa =\kappa (\ell )$ and right plot presents $\kappa$ as a numerical parameter which is sufficiently small

Furthermore, the second-order constitutive stress tensor with respect to Eq. 18 reads

20 $$\begin{aligned}&{\varvec{\sigma }}(\varvec{\varepsilon },d ):=\frac{\partial w_{\mathrm{bulk}}(\varvec{\varepsilon }, d)}{\partial {\varvec{\varepsilon }}} =g(d_+)\frac{\partial {\widetilde{\Psi }}^{+}}{\partial \varvec{\varepsilon }}\nonumber \\&\qquad \quad \;\quad +\frac{\partial {\widetilde{\Psi }}^{-}}{\partial \varvec{\varepsilon }} =g(d_+)~{\varvec{{\widetilde{\sigma }}}^{+}}+{\varvec{{\widetilde{\sigma }}}^{-}}, \end{aligned}$$

with

21 $$\begin{aligned}&\varvec{{\widetilde{\sigma }}}^{\pm }(\varvec{\varepsilon }) :=K~I_1^{\pm }(\varvec{\varepsilon }) -2\mu ~\Big (\frac{1}{3}I_1^{\pm }(\varvec{\varepsilon }) \mathbf{I } \nonumber \\&\quad - 2 \varvec{\varepsilon }_\pm \Big ) \quad \text {with}\quad K>0\quad \text {and}\quad \mu >0. \end{aligned}$$

### Crack driving forces for brittle failure

Following [33, 34], we determine the crack driving state function to couple between two PDEs. Hence, crack driving state function acts as a right hand side for the phase-field equation. To formulate the crack driving state function, we consider the crack irreversibility condition, which is the inequality constraint $\dot{d} \le 0$ imposed on our variational formulation. The first variation of the total pseudo-energy density with respect to the crack phase-field given in (17) reads

22 $$\begin{aligned}&- \delta _{d} w(\varvec{\varepsilon }, d, \nabla d)=(\kappa -1)2d_+ \big [{\widetilde{\Psi }}^{+} \big ]\nonumber \\&- G_c \delta _d\gamma _\ell (d, \nabla d) \ge 0. \end{aligned}$$

Herein, the functional derivative of $\gamma _l(d,\nabla d)$ with respect to *d* is

23 $$\begin{aligned} \int _\Omega \delta _{d} \gamma _{\ell }(d,\nabla d) \mathrm {d}{\textit{\textbf{x}}}=\int _\Omega \frac{1}{\ell }[(d-1)-\ell ^2 \Delta d] \mathrm {d}{\textit{\textbf{x}}}. \end{aligned}$$

Maximization the inequality given in Eq. 22 with respect to the time history $s\in [0,t_n]$ reads

24 $$\begin{aligned} (\kappa -1)2d_+\max _{s \in [0,t_n]} \big [{\widetilde{\Psi }}^{+} \big ] =G_c \delta _d\gamma _\ell (d, \nabla d). \end{aligned}$$

We multiply Eq. 24 by $\frac{l}{G_c}$. Then Eq. 24 can be restated as

25 $$\begin{aligned} (\kappa -1)2d_+\mathcal {H}= & {} \ell \delta _d\gamma _l \quad \text {if} \quad \nonumber \\ \mathcal {H}:= & {} \max _{s\in [0,t_n]}{\widetilde{D}} \quad \text {with} \quad {\widetilde{D}}:=\frac{\ell {{\widetilde{\Psi }}}^{+}}{G_c}. \end{aligned}$$

Here, $\mathcal {H}:=\mathcal {H}(\varvec{\varepsilon },t)$ denotes a positive crack driving force that is used as a history field from initial time up to the current time. Note that the crack driving state function ${\widetilde{D}}$ is affected by the length-scale parameter $\ell$ and hence depends on the regularization parameter.

#### Formulation 2.3

(Final Euler–Lagrange equations) Let us assume that $K>0$, $\mu >0$ are given as well as the initial condition $\textit{\textbf{u}}_0=\textit{\textbf{u}}(\textit{\textbf{x}},0)$ and $d_0=d(\textit{\textbf{x}},0)$. For the loading increments $n \in \{1,2,\ldots , N\}$, find $\textit{\textbf{u}}:=\textit{\textbf{u}}^n\in V$ and $d:=d^n\in W$ such that

27 $$\begin{aligned} \begin{aligned}&{{\mathcal {E}}}_{\textit{\textbf{u}}}(\textit{\textbf{u}}, d_+;\delta \textit{\textbf{u}})= \int _\Omega g(d_+) {\varvec{{\widetilde{\sigma }}}^{+}_{\varvec{\varepsilon }}}(\textit{\textbf{u}}): {\varvec{\varepsilon }}(\delta \textit{\textbf{u}})\,\mathrm {d}{\textit{\textbf{x}}}\\&\quad + \int _\Omega {\varvec{{\widetilde{\sigma }}}^{-}_{\varvec{\varepsilon }}(\textit{\textbf{u}})}: {\varvec{\varepsilon }}(\delta \textit{\textbf{u}})\,\mathrm {d}{\textit{\textbf{x}}}\\&\quad -\int _{\partial _N\Omega } {\varvec{{\bar{\tau }}}} \cdot {\delta \textit{\textbf{u}}}\,\mathrm {d}s= 0 \qquad \forall {\delta \textit{\textbf{u}}}\in {\textit{\textbf{V}}}, \\&{{\mathcal {E}}}_d(\textit{\textbf{u}},d;\delta d) =(1-\kappa )\int _\Omega 2d_+ \mathcal {H} \delta d \,\mathrm {d}{\textit{\textbf{x}}}\\&\quad + \int _\Omega \Big ((d-1) \delta d + \ell ^2 \nabla d\cdot \nabla \delta d\Big )\mathrm {d}\mathbf{x }= 0 \qquad \forall \delta d\in W. \end{aligned} \end{aligned}$$

The multi-field problem given in Formulation (2.3) depending on $\textit{\textbf{u}}$ and *d* implies alternately fixing $\textit{\textbf{u}}$ and *d*, which is a so called alternate minimization scheme, and then solving the corresponding equations until convergence. The alternate minimization scheme applied to the Formulation (2.3) is summarized in Algorithm 1.
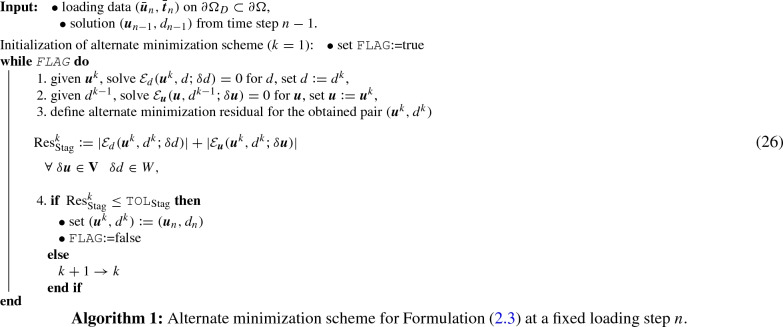


### The influence of the $\kappa$ on the stress-strain curve

In this part, the influence of the $\kappa$ on the stress-strain curve is taken into account. Following [26], the homogeneous solution at the quasi-static stationery state of the phase-field partial differential equation in the loading case takes the following form

28 $$\begin{aligned} d_{\mathrm{homo}}=\frac{1}{1+2(1-\kappa ){\widetilde{D}}} \;\in [0,1], \end{aligned}$$

which results from the free Laplacian operator $\Delta (\bullet )=0$ assumption in Eq. 24 without any source terms (zero left-hand sides). Here, the crack driving state function ${\widetilde{D}}$ is given in Eq. 25. Because, we are in the elastic limit, prior to the onset of fracture, then no split is considered. We now aim to relate a stress state $\sigma$ with the isotopic phase-field formulation. To do so, a non-monotonous function in the one-dimensional setting for the degrading stresses takes the following form by

29 $$\begin{aligned} \sigma= & {} g(d){\widetilde{\sigma }} =\left( \frac{(1-\kappa )}{\left( 1+2(1-\kappa ){\widetilde{D}}\right) ^{2}}+\kappa \right) ~ E\varepsilon . \end{aligned}$$

To see the influence of the $\kappa$ in Eq. 29, the concrete material is considered. Following, [35] for a concrete material which has a brittle response, a typical values for material parameters reads,

30 $$\begin{aligned} E=29\,\mathrm{GPa},\quad \sigma _c=4.5\,\mathrm{MPa} \quad \text {and} \quad G_c=70\,\mathrm{N/m}. \end{aligned}$$

We set $\ell =0.0105~\mathrm{m}$. Thus, we can do a plot for the stress-strain curve through Eq. 29 by considering the material set given above. Figure 3 shows the effect of stress state for different strain loading. The black curve represents the stress-strain curve while $\kappa =0$. Evidently, it can be grasped through Fig. 3 with $\kappa =0$ the $\sigma _c$ is exactly $\sigma _c=4.5\,\mathrm{MPa}$ as it is required for the concrete material, see [35]. If we consider $\kappa \ne 0$ as a function of characteristic length-scale, see Fig. 3 left, we can observe a good agreement with $\kappa =0$ up to the peak point while after some strain value it becomes different as $\kappa$ changes. Unfortunately, we can not observe any converged response if we consider $\kappa$ as a function of $\ell$. In contrast, if we chose $\kappa$ sufficiently small, see Fig. 3 right, as much as $\kappa$ reduced, in here less than $\kappa \le 10^{-4}$, we observed a very identical response with $\kappa =0$, thus it behaves as numerical parameters rather than material parameters.

## Stochastic model for Bayesian inversion

In this section, we explain how we use Bayesian inversion to identify parameters. Then, we introduce a computationally effective numerical technique to estimate the unknown parameters.

In the phase-field model, the uncertainties arise from the elasticity parameters including the shear modulus $\mu$ and the bulk modulus *K* as well as Griffith’s critical elastic energy release rate (material stiffness parameter) $G_c$, which are assumed to be random fields. Specifically, we represent the parameters uncertainty (spatial variability) by a spatially-varying log-normal random field.

The Karhunen-Loéve expansion (KLE) expansion method is used to reduce the dimensionality of the random field. The field $\Theta$ representing the elasticity parameters and the energy release rate can be characterized by its expectation and covariance using the expansion. Considering the probability density function $\mathbb {P}$, the covariance function is

31 $$\begin{aligned}&{\text {Cov}}_{\Theta }(\textit{\textbf{x}},\mathbf{y} )=\int _{\Omega }\left( \Theta (\textit{\textbf{x}},\omega )-\Theta (\textit{\textbf{x}})\right) \left( \Theta (\textit{\textbf{y}},\omega )-\Theta (\textit{\textbf{y}})\right) \,\text {d}\mathbb {P}(\omega ), \end{aligned}$$

which leads to the KL-expansion

32 $$\begin{aligned} \Theta (\textit{\textbf{x}},\omega )=\bar{\Theta }(\textit{\textbf{x}})+\sum _{n=1}^{\infty }\sqrt{\psi _n }k_n(\textit{\textbf{x}}) \xi _n (\omega ). \end{aligned}$$

Here the first term is the mean value, $k_n$ are the orthogonal eigenfunctions, $\psi _n$ are the corresponding eigenvalues of the eigenvalue problem [36]

33 $$\begin{aligned} \int _{D}\text {Cov}_{\Theta } (\textit{\textbf{x}},\textit{\textbf{y}})k_n(\textit{\textbf{y}})~ d\textit{\textbf{y}}=\psi _n k_n(\textit{\textbf{x}}), \end{aligned}$$

and the $\lbrace \xi _n(\omega )\rbrace$ are mutually uncorrelated random variables satisfying

34 $$\begin{aligned} \mathbb {E}[\xi _n]=0,\mathbb {E}[\xi _n \xi _m]=\delta _{nm}, \end{aligned}$$

where $\mathbb {E}$ indicates the expectation of the random variables.

The infinite series can be truncated to a finite series expansion (i.e., an $N_\mathrm {KL}$-term truncation) by [36]

35 $$\begin{aligned} \Theta (\textit{\textbf{x}},\omega )=\bar{\Theta }(\textit{\textbf{x}})+\sum _{n=1}^{N_{_{\text {KL}}}}\sqrt{\psi _n}k_n(\textit{\textbf{x}})\xi _n(\omega ). \end{aligned}$$

For the Gaussian random field, we employ an exponential covariance kernel as

36 $$\begin{aligned} {\text {Cov}}_{\Theta } (\textit{\textbf{x}},\textit{\textbf{y}})=\sigma ^2\exp \left( -\frac{\Vert \textit{\textbf{x}}-\textit{\textbf{y}}\Vert }{\zeta }\right) , \end{aligned}$$

where $\zeta$ is the correlation length as well as $\sigma$ is the standard deviation.

For a random field, we describe the parameters using a KL-expansion. Considering the Gaussian field $\xi (\textit{\textbf{x}})$, a log-normal random field can be generated by the transformation ${\tilde{\xi }}(\textit{\textbf{x}})=\exp (\xi (\textit{\textbf{x}}))$. For instance, for the parameter *K*, the truncated KL-expansion can be written as

37 $$\begin{aligned} {\tilde{\xi }}_{{K}} (\textit{\textbf{x}},\omega )=\exp \left( \bar{\xi _{{K}}}(\textit{\textbf{x}},\omega )+\sum _{n=1}^{N}\sqrt{\psi _n}k_n(\textit{\textbf{x}})\xi _n(\omega )\right) . \end{aligned}$$

### Bayesian inference

We consider Formulation 2.3 as the forward model $\mathbf{y} =\mathcal {G}(\,\Theta \,(\textit{\textbf{x}}))$, where $\mathcal {G}:L^2(\Omega )\rightarrow L^2(\Omega )$. The forward model explains the response of the model to different influential parameters $\Theta$ (here $\mu$, *K*, and $G_c$). We can write the statistical model in the form [37]

38 $$\begin{aligned} \mathcal {M}=\mathcal {G}(\,\Theta )+\varepsilon , \end{aligned}$$

where $\mathcal {M}$ indicates a vector of observations (e.g., measurements). The error term $\varepsilon$ arises from uncertainties such as measurement error due experimental situations. More precisely, it is due to the modeling and the measurements and is assumed to have a Gaussian distribution of the form $\mathcal {N}(0,H)$ with known covariance matrix *H*. The error is independent and identically distributed and is independent from the realizations. Here, for sake of simplicity, we assume $H=\sigma ^2I$ (for a positive constant $\sigma ^2$).

For a realization $\theta$ of the random field $\Theta$ corresponding to a realization *m* of the observations $\mathcal {M}$, the posterior distribution is given by

39 $$\begin{aligned} \pi (\theta |m)=\frac{\pi (m|\theta )\pi _0(\theta )}{\pi (m)}=\frac{\pi (m|\theta )\pi _0(\theta )}{\int _{W_m}\pi (m|\theta )\pi _0(\theta )\,d\theta }, \end{aligned}$$

where $\pi _0(\theta )$ is the prior density (prior knowledge) and ${W_m}$ is the space of parameters *m* (the denominator is a normalization constant) [38]. The likelihood function can be defined as [37]

40 $$\begin{aligned} \pi (m|\theta ):=\frac{1}{(2\pi \sigma ^2)^{{\bar{n}}/2}}\exp \left( -\sum _{n=1}^{{\bar{n}}} \frac{(m_n-\mathcal {G}(\theta ))^2}{2\sigma ^2} \right) . \end{aligned}$$

**Fig. 4:**
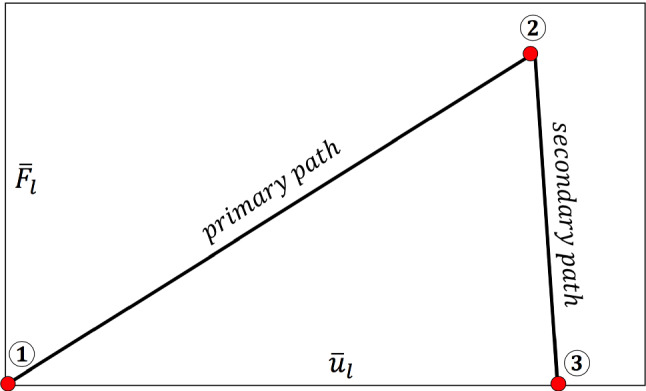
The schematic of load-deflection response for the failure process including primary path (prior to the crack initiation, i.e., between point 1 and 2) and secondary path (during crack propagation, i.e., between point 2 and 3)

As an essential characteristic of the phase-field model, the load-displacement curve (i.e., the global measurement) in addition to the crack pattern (i.e., the local measurement) are appropriate quantities to show the crack propagation as a function of time. Figure 4 indicates the load-displacement curve during the failure process. Three major points are the following.

① **First stable position**. This point corresponds to the stationary limit such that we are completely in elastic region ($d(\textit{\textbf{x}},0)=1 \;\forall \textit{\textbf{x}}\in \Omega \backslash \mathcal {C}$).

② **First peak point**. Prior to this point crack nucleation has occurred and now we have crack initiation. Hence, this peak point corresponds to the critical load quantity such that the new crack surface appears (i.e., there exist some elements which have some support with $d=0$).

③ **Failure point**. At this point, failure of the structure has occurred and so increasing the load applied to the material will not change the crack surface anymore.

The interval between point 1 and point 2 in Fig. 4 typically refers to the primary path where we are almost in the elastic region. The secondary path (sometimes referred to as the softening damage path) starts with crack initiation occurring at point 2. The whole process recapitulates the load-deflection curve in the failure process.

The main aim of solving the inverse problem followed here is to determine the random field $\Theta$ to satisfy (38). We strive to find a posterior distribution of suitable values of the parameters $\mu$, *K*, and $G_c$ in order to match the simulated values (arising from (27)) with the observations. The distribution provides all useful statistical information about the parameter.

#### Remark 3.1

Note that the principal parameters *h*, $\kappa$, and $\ell$ are mathematically linked in Formulation  2.3. Here, we use $\ell =2h$ and $\kappa$ is sufficiently small which is compatible with Sect. 2.7. In Sect. 5, the values of $\kappa$ in the computations will be specified. Further, a sufficiently small *h* is chosen to obtain the reference solution.

The crack pattern is a time-dependent process (more precisely in a quasi-static regime, the cracking process is load-dependent), i.e., after initiation it is propagated through time. In order to approximate the parameters precisely, we estimate the likelihood during all time steps. Therefore, the posterior distribution maximizes the likelihood function for all time steps, and therefore we have an exacter curve for all crack nucleation and propagation times.

MCMC is a suitable technique to calculate the posterior distribution. When the parameters are not strongly correlated, the MH algorithm [39] is an efficient computational technique among MCMC methods. We propose a new candidate (so-called $\theta$, i.e., a value of $(\mu ,{K},G_c)$) according to a proposal distribution (for instance uniform or normal distributions) and calculate its acceptance/rejection probability. The ratio indicates how likely the new proposal is with regard to the current sample. In other words, by using the likelihood function (40), the ratio determines whether the proposed value is accepted or rejected with respect to the observation (here the solution of Formulation 2.3 with a very fine mesh). As mentioned, fast convergence means that the parameters are fully correlated. A summary of the MH algorithm is given below.
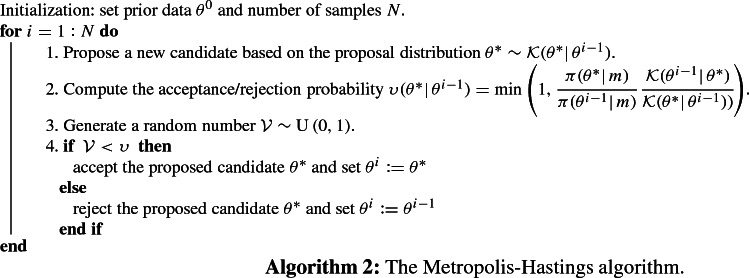


**Fig. 5:**
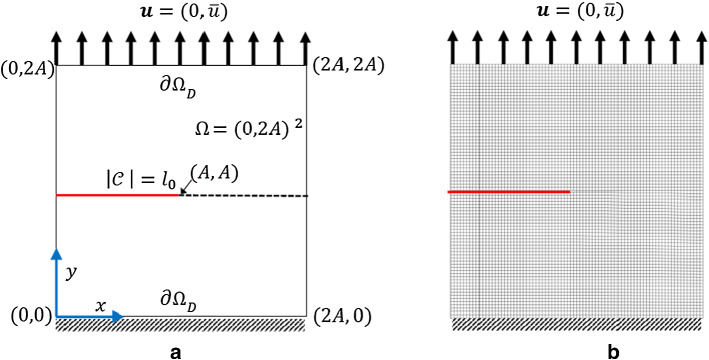
Schematic of SENT (Example 1) (left) and its corresponding mesh with $h=1/80$ (right)

**Fig. 6:**
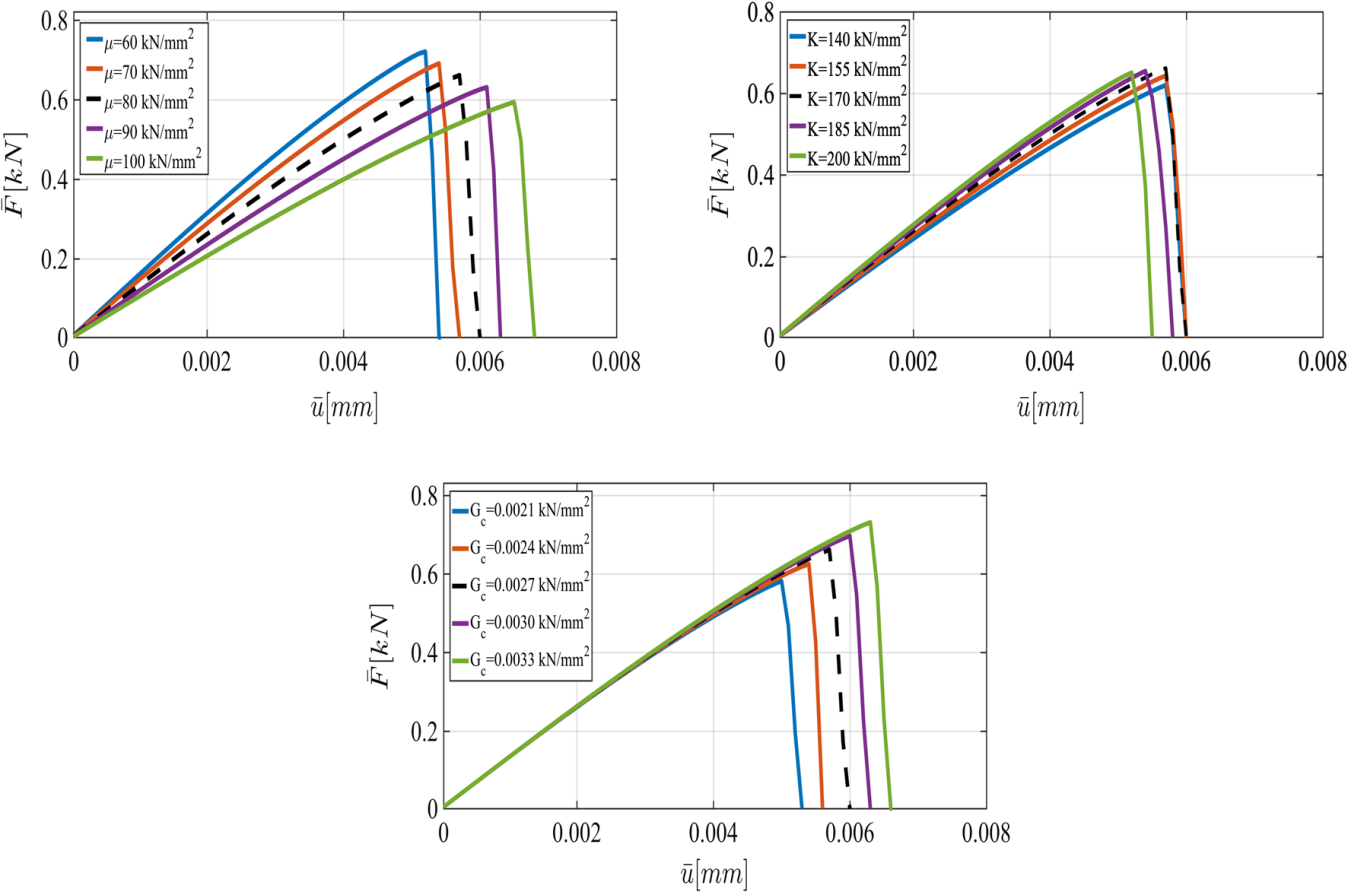
The load-displacement curve for different values of $\mu$ (top left), *K* (top right) and $G_c$ (bottom) in the SENT example (Example 1)

**Fig. 7:**
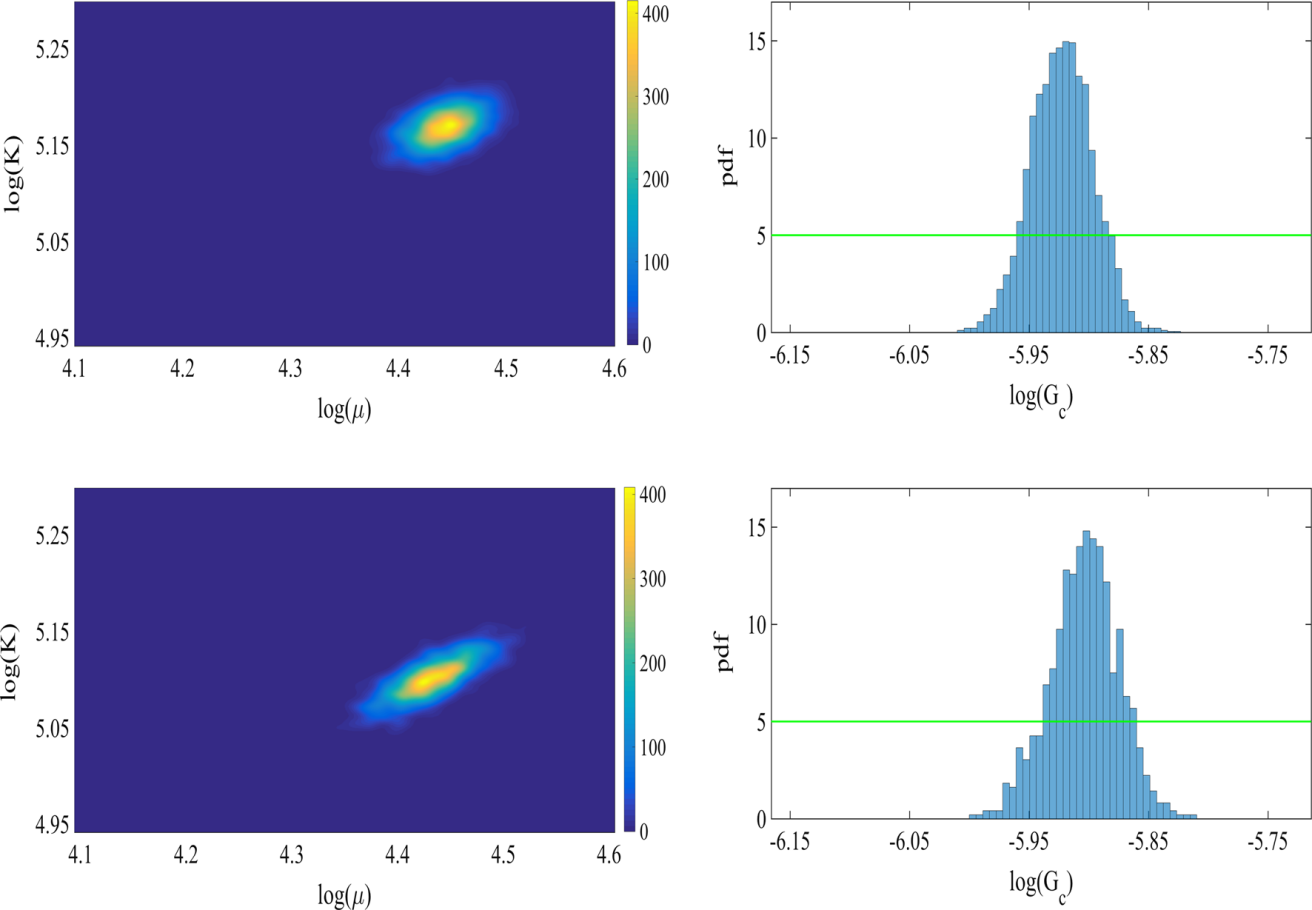
Left: the joint probability density of the elasticity parameters. Right: the prior (green line), and the normalized probability density function (pdf) of $G_c$ for the SENT example. Here we compare the distributions obtained by the one-dimensional Bayesian inversion (first row) and the three-dimensional Bayesian inversion (second row)

**Table 1:** The mean values of the posterior distributions obtained by one-dimensional and three-dimensional Bayesian inversion in addition to their acceptance ratios for the SENT (Example 1)

	One-dimensional		Three-dimensional	
	Mean ($\mathrm {kN/mm^2}$)	Ratio (%)	Mean ($\mathrm {kN/mm^2}$)	Ratio (%)
$\mu$	84.2	27	85.1	28
*K*	176.1	27	175.3	28
$G_c$	0.00272	29.1	0.00268	28.6

The units are in $\mathrm {kN/mm^2}$

## Bayesian inversion for phase-field fracture

In this key section, we combine the phase-field algorithm from Sect. 2 with the Bayesian framework presented in Sect. 3.

First, we define two sampling strategies as follows:

- *One-dimensional* Bayesian inversion. We first use *N* samples (according to the proposal distribution) and extract the posterior distribution of the first set e.g., ($\mu ^*$, $K^*$) where other parameter is according to the mean value. Then obtained information is used to estimate the posterior distribution of next unknown (i.e., $G_c^*$). In order to employ the estimated values, the exponential of the estimated parameters is used in the AT-2 model (see Algorithm 2).
- $\textit{Multi-dimensional}$ Bayesian inversion. A three-dimensional candidate $(\mu ^*,K^*,G_c^*)$ is proposed and the algorithm computes its acceptance/rejection probability.

To make the procedures more clarified we explain the multi-dimensional approach in Algorithm 3. Clearly, for the one-dimensional setting; for each parameter (e.g., $\theta ^*={(\mu ^*,K^*)}$), it can be reproduced separately. We will study both techniques in the first example and the more efficient method will be used for other simulations.
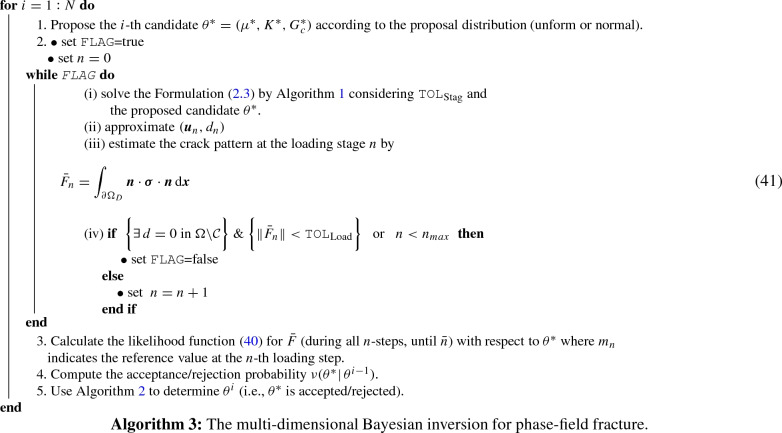
 Here, $n_{\mathrm{max}}$ is the sufficiently large value that is set by the user. Also, $\texttt {tol}_\mathrm {load}$ is a sufficiently small value to guarantee that the crack phase-field model reached to the material failure time. Note, in part (iv) for the while-loop step, the criteria $\Vert {\bar{F}}_n \Vert <\texttt {tol}_\mathrm {load}$ in the secondary path (i.e., during crack propagation state) guarantees that reaction force under imposed Dirichlet boundary surface is almost zero. Hence, no more force exists to produce a fractured state. We now term this as a complete failure point. But, in some cases, e.g., shear test as reported in [31], by increasing the monotonic displacement load, ${\bar{F}}_n$ is not reached to zero. For this type of problem, if $n<n_{\mathrm{max}}$ holds, then phase-field step (i.e., while-loop step) in Algorithm 3 will terminate.

The physical aim of using Bayesian inversion in phase-field fracture is adjusting the effective parameters to fit the solution with the reference values (see Remark 3.1). With (future) experiments (experimental load-displacement until the failure point), these can be used as reliable reference values.

**Fig. 8:**
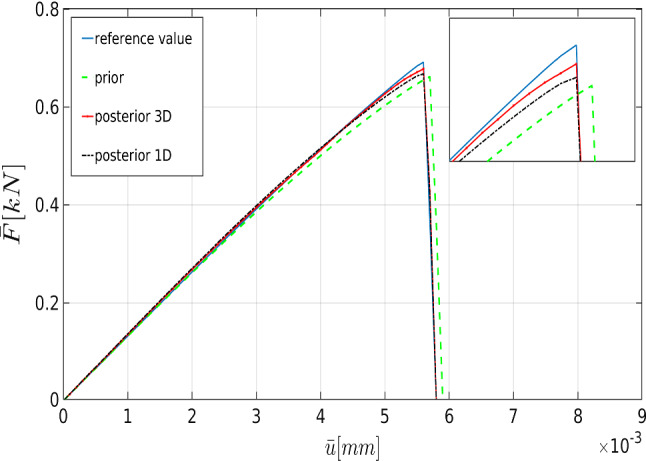
The load-displacement curve for the one-dimensional (black) and three-dimensional (red) posterior distributions in addition to the ones for the prior distribution (green) and the reference value (blue) for the SENT example (Example 1) with $h=1/160$

**Fig. 9:**
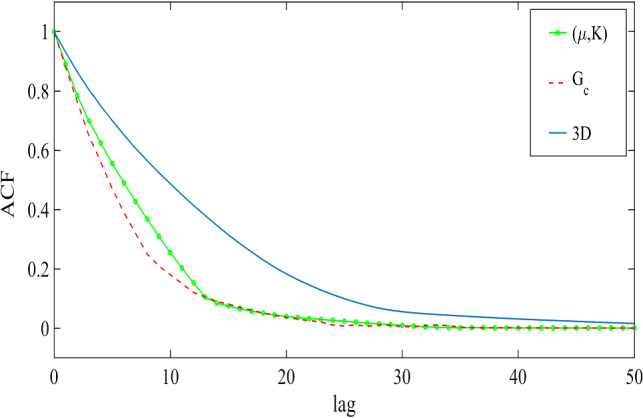
The autocorrelation function for one- and multidimensional Bayesian inference in the SENT example

## Numerical examples

In this section, we consider three numerical test problems to determine the unknown parameters using given Bayesian inference. Specifically, we propose:

- Example 1 the single edge notch tension (SENT) test;
- Example 2 double edge notch tension (DENT) test;
- Example 3 tension test with two voids.

The observations can be computed by very fine meshes (here the reference values) as an appropriate replacement of the measurements (see Remark 3.1). Regarding the observational noise, $\sigma ^2=1\times 10^{-3}$ is assumed. The main aim here is to estimate the effective parameters ($\mu$, ${K}$, and $G_c$) in order to match the load-displacement curve with the reference value. To characterize the random fields, we can use the KL-expansion with $N_{_{\text {KL}}}=100$ and the correlation length $\zeta =2$ as well.

In all examples, the phase-field parameters set by $\kappa = 10^{-8}$, and regularized length scale $\ell = 2{h}$ (respecting the condition $h < l$). The stopping criterion for the iterative Newton method scheme, i.e. the relative residual norm that is42is chosen to $\texttt {Tol}_\texttt {N-R}=10^{-8}$. Here, $\textit{\textbf{R}}$ indicates a discretized setting of weak forms described in Formulation (2.3). Regarding alternate minimization scheme we set $\texttt {TOL}_\mathrm {Stag}=10^{-4}$ for all numerical examples and $\texttt {TOL}_\mathrm {Load}=10^{-3}$ is chosen to guarantee that we solve the model only until the material failure time. In the examples, the random fields modeled as a log-normal random field. For the numerical simulations, all variables are discretized by first-order quadrilateral finite elements.

**Fig. 10:**
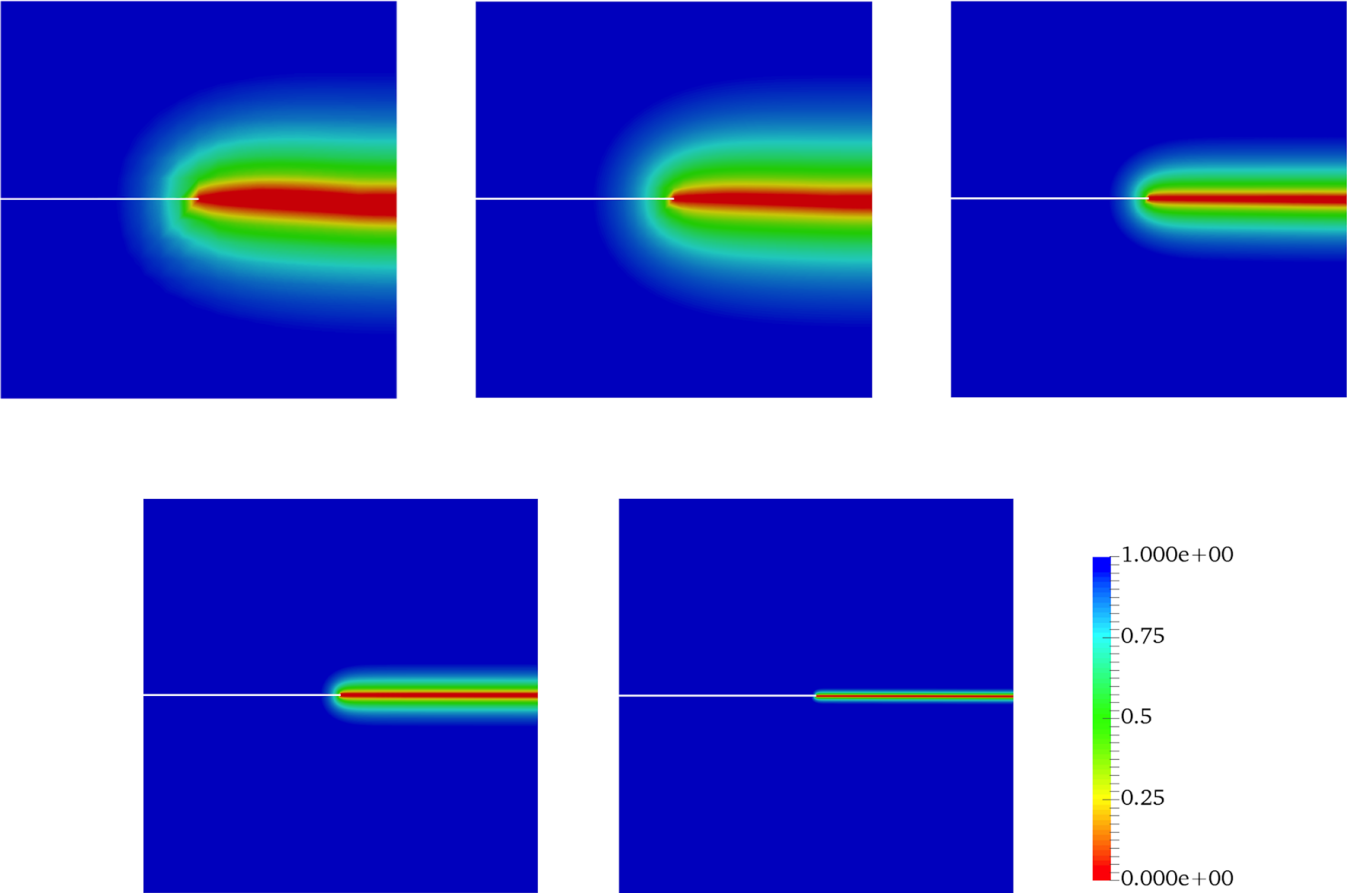
The effect of the mesh size on the crack propagation in the SENT example (Example 1). The mesh sizes are (from the left) $h=1/20$, $h=1/40$, $h=1/80$, $h=1/160$, and $h=1/320$ (the reference). The effective parameters are chosen according to the prior values

### Example 1: The single edge notch tension (SENT) test

This example considers the single edge notch tension. The specimen is fixed at the bottom. We have traction-free conditions on both sides. A non-homogeneous Dirichlet condition is applied at the top. The domain includes a predefined single notch (as an initial crack state imposed on the domain) from the left edge to the body center, as shown in Fig. 5a. We set $A=0.5\;\mathrm {mm}$ hence $\Omega =(0,1)^2 \mathrm {mm}^2$, hence the predefined notch is in the $y=A$ plane and is restricted to $0\le |\mathcal {C}|\le A$. This numerical example is computed by imposing a monotonic displacement ${\bar{u}}=1\times 10^{-4}$ at the top surface of the specimen in a vertical direction. The finite element discretization corresponding to $h=1/80$ is indicated in Fig. 5b.

For the shear modulus, we assume the variation range $(60\,\mathrm {kN/mm^2},100\,\mathrm {kN/mm^2})$. Regarding the the bulk modulus *K*, the parameter varies between $140\,\mathrm {kN/mm^2}$ and $200\,\mathrm {kN/mm^2}$. Finally, we consider the interval between $2.1\times 10^{-3}\,\mathrm {kN/mm^2}$ and $3.3\times 10^{-3}\,\mathrm {kN/mm^2}$ for $G_c$. Furthermore, we assume that in this example, the variables are spatially constant random variables (they are not random fields).

**Fig. 11:**
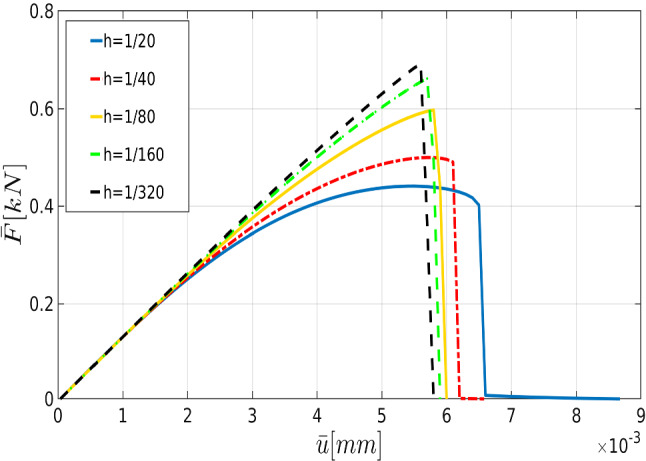
The load-displacement curve in the SENT example (Example 1) for different mesh sizes, where the parameters are chosen according to the prior

We solved the PDE model (Formulation 2.3) with $\mu =80\,\mathrm {kN/mm^2}$, $K=170\,\mathrm {kN/mm^2}$, and $G_c=2.7\times 10^{-3}\,\mathrm {kN/mm^2}$ [31] and the displacement during the time (as the reference solution) with $h=1/320$ was obtained. The main goal is to obtain the suitable values of $\mu ,~{K}$, and $G_c$ such that the simulations match the reference value.

For this example, we use a uniformly distributed prior distribution and the uniform proposal distribution

43 $$\begin{aligned} \mathcal {K}(\theta \rightarrow \theta ^*):=\frac{1}{\theta _2-\theta _1}\chi [\theta _1,\theta _2](\theta ), \end{aligned}$$

where $\chi$ indicates the characteristic function of the interval $[\theta _1,\theta _2]$ (where $\theta$ denotes a set of parameters).

First, we describe the effect of each parameter on the displacement. As the elasticity constants (i.e., $\mu$ and ${K}$) become larger, the material response becomes stiffer; crack initiation takes longer to occur. Additionally, a larger crack release energy rate (as an indicator for the material resistance against the crack driving force) delays crack nucleation and hence crack dislocation. All these facts are illustrated in Fig. 6.

**Fig. 12:**
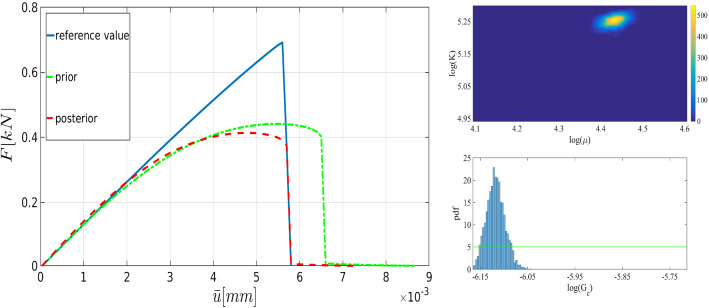
The load-displacement curves for the reference, prior, and posterior values (left panel) for $h=1/20$ in the SENT example (Example 1). The joint and marginal posterior distributions of the effective parameters are shown in the right panel

**Fig. 13:**
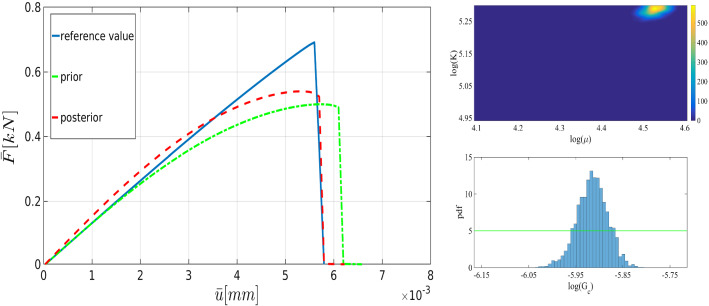
The load-displacement curves for the reference, prior, and posterior values (left panel) for $h=1/40$ in the SENT example (Example 1). The joint and the marginal posterior distributions of the effective parameters are shown in the right panel

**Fig. 14:**
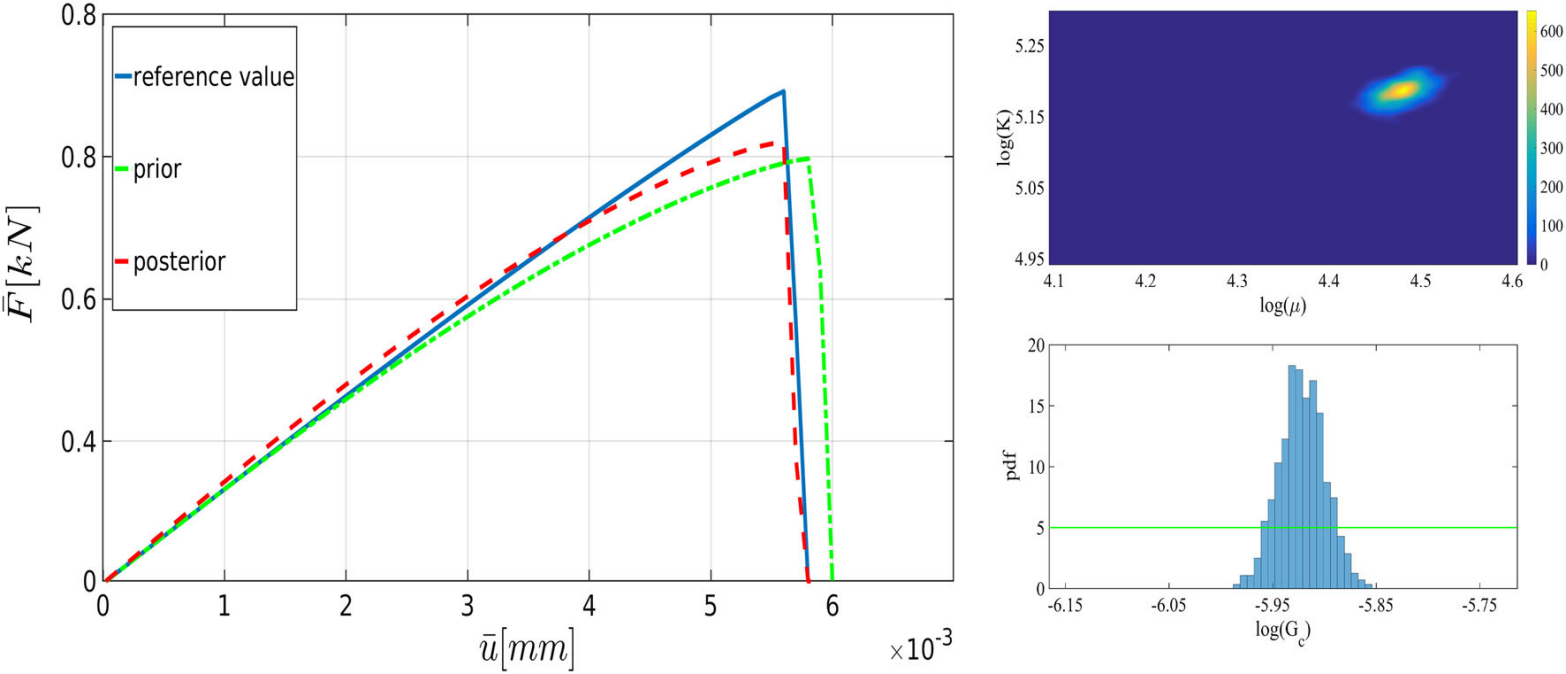
The load-displacement curves for the reference, prior, and posterior values (left panel) for $h=1/80$ in the SENT example (Example 1). The joint and the marginal posterior distributions of the effective parameters are shown in the right panel

The joint probability density of the elasticity parameters and the marginal probability of the posterior are shown in Figure 7 including one- and three-dimensional Bayesian inversions. The mean values of the distributions are $\mu =84.2\,\mathrm {kN/mm^2}$ and $\mu =85.1\,\mathrm {kN/mm^2}$ are obtained for the shear modulus. Here an acceptance rate of 27% is obtained. Regarding the material stiffness parameter $G_c$, the acceptance rates are near 29%. The values are summarized in Table 1.

To verify the parameters obtained by the Bayesian approach, we solved the forward model using the mean values of the posterior distributions. Figure 8 shows the load-displacement diagram according to prior and posterior distributions. As expected, during the nucleation and propagation process, using Bayesian inversion results in better agreement (compared to the prior). Furthermore, a better estimation is achieved by simultaneous *multi-dimensional* Bayesian inversion. From now onward, this approach will be used for Bayesian inference.

**Table 2:** The mean of thel posterior distributions of $\mu$, ${K}$, and $G_c$ in the SENT example (Example 1) for $h=1/20$, $h=1/40$, and $h=1/80$. All units are in $\mathrm {kN/mm^2}$

	$\mu$	Rate (%)	${K}$	Rate (%)	$G_c$	Rate (%)
$h=1/20$	83,9	21	190.4	21	0.00221	23
$h=1/40$	92.4	20	197.2	20	0.00271	26
$h=1/80$	88.1	27	178.4	27	0.00266	28.1

**Table 3:** The elapsed CPU time for the estimation of the load-displacement diagram (with the reference values) until the failure point in SENT

Mesh size	$h=1/20$	$h=1/40$	$h=1/80$	$h=1/160$	$h=1/320$
CPU time [s]	10	23	1428	4810	15 854

#### The convergence of MCMC

A customary method to assess the convergence of the MCMC is the calculation of its autocorrelation. The lag-$\tau$ autocorrelation function (ACF) $R:\mathbb {N}\rightarrow [-1,1]$ is defined as

$$\begin{aligned} R(\tau ):=\frac{\sum _{n=1}^{N-\tau } (\theta _n -\bar{\theta })(\theta _{n+\tau } -\bar{\theta }) }{ \sum _{n=1}^{N}\left( \theta _n-\bar{\theta }\right) ^2}=\frac{\text {cov}(\theta _n,\theta _{n+\tau })}{\text {var}(\theta _n)}, \end{aligned}$$

where $\theta _n$ is the *n*-th element of the Markov chain and $\bar{\theta }$ indicates the mean value. For the Markov chains, $R(\tau )$ is positive and strictly decreasing. Also, a rapid decay in the ACF indicates the samples are not fully correlated and mixing well. Figure 9 shows the convergence of the MCMC where the elasticity parameters and the crirical elastic energy. Also, we estimated the convergence observed in the multi-dimensional approach. As expected, the multi-dimensional approach converges slower than the one-dimensional one.

**Fig. 15:**
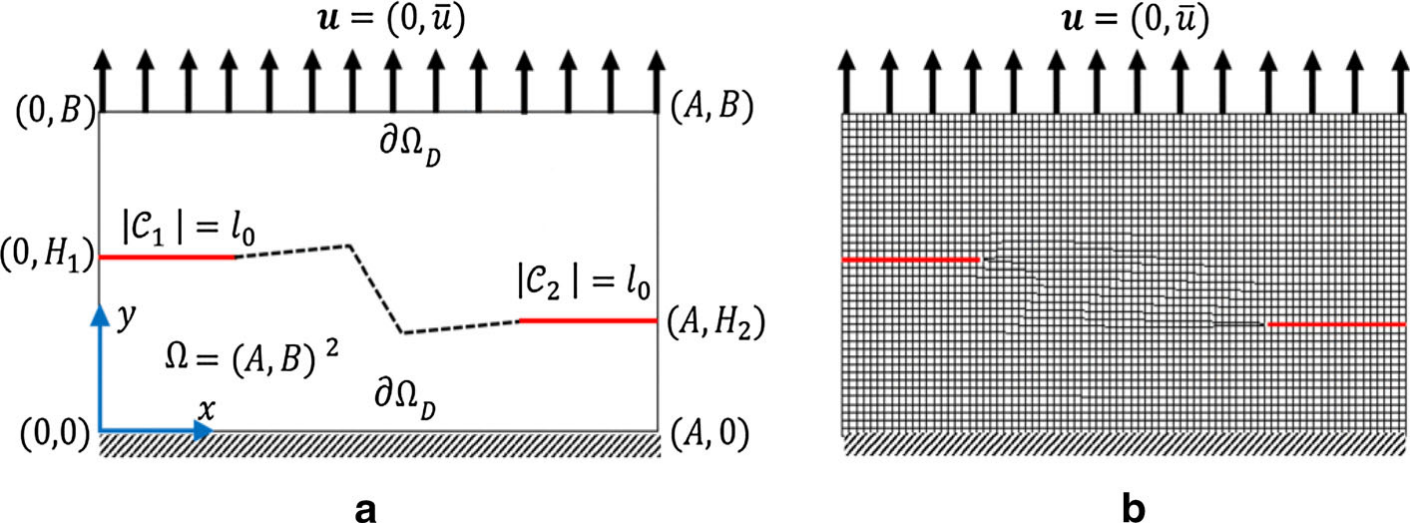
Schematic diagram for the DENT example (left) and its corresponding mesh with $h=1/80$ (right)

**Fig. 16:**
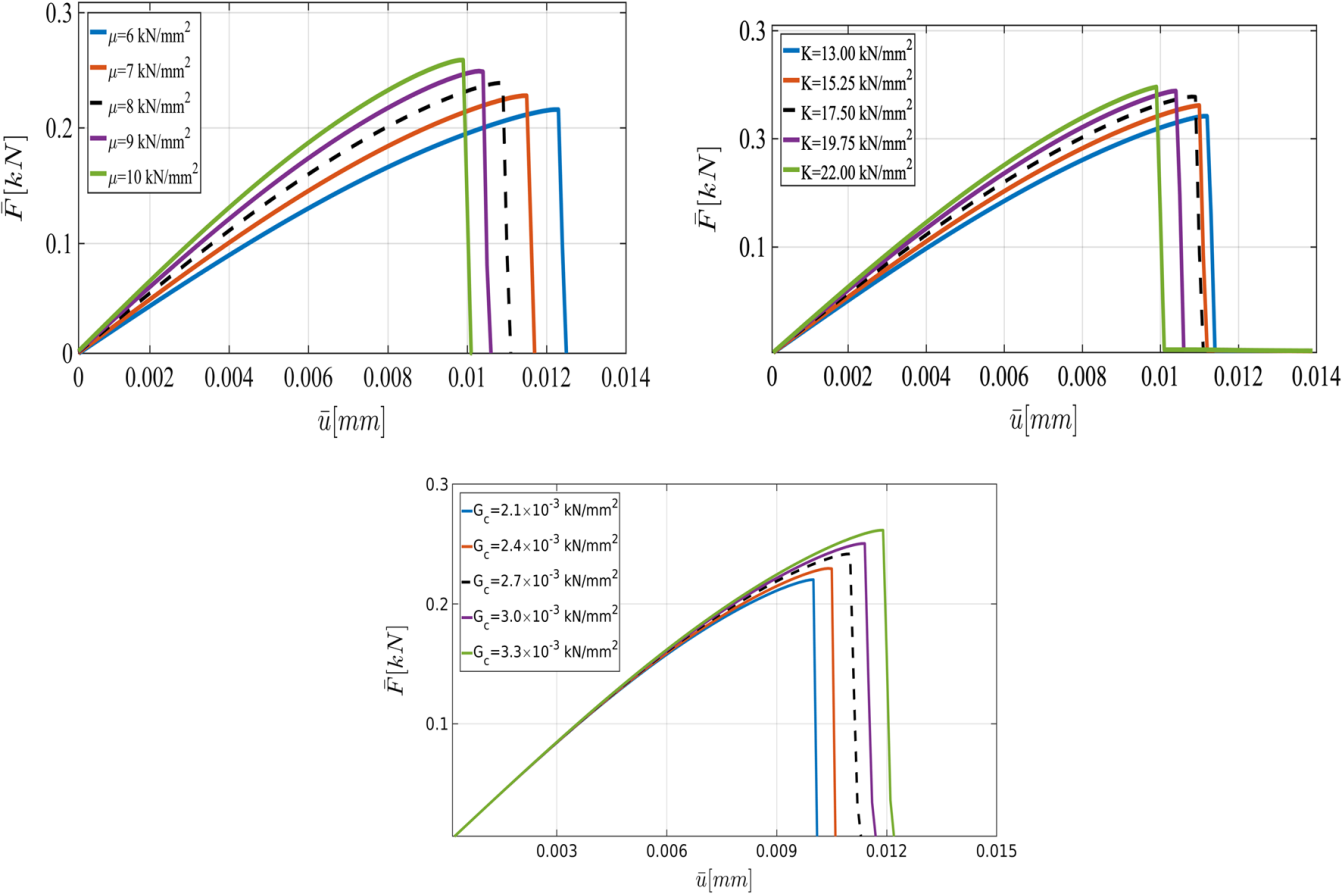
The load-displacement curve for different values of $\mu$ (top left), *K* (top right) and $G_c$ (bottom) for the DENT example

**Fig. 17:**
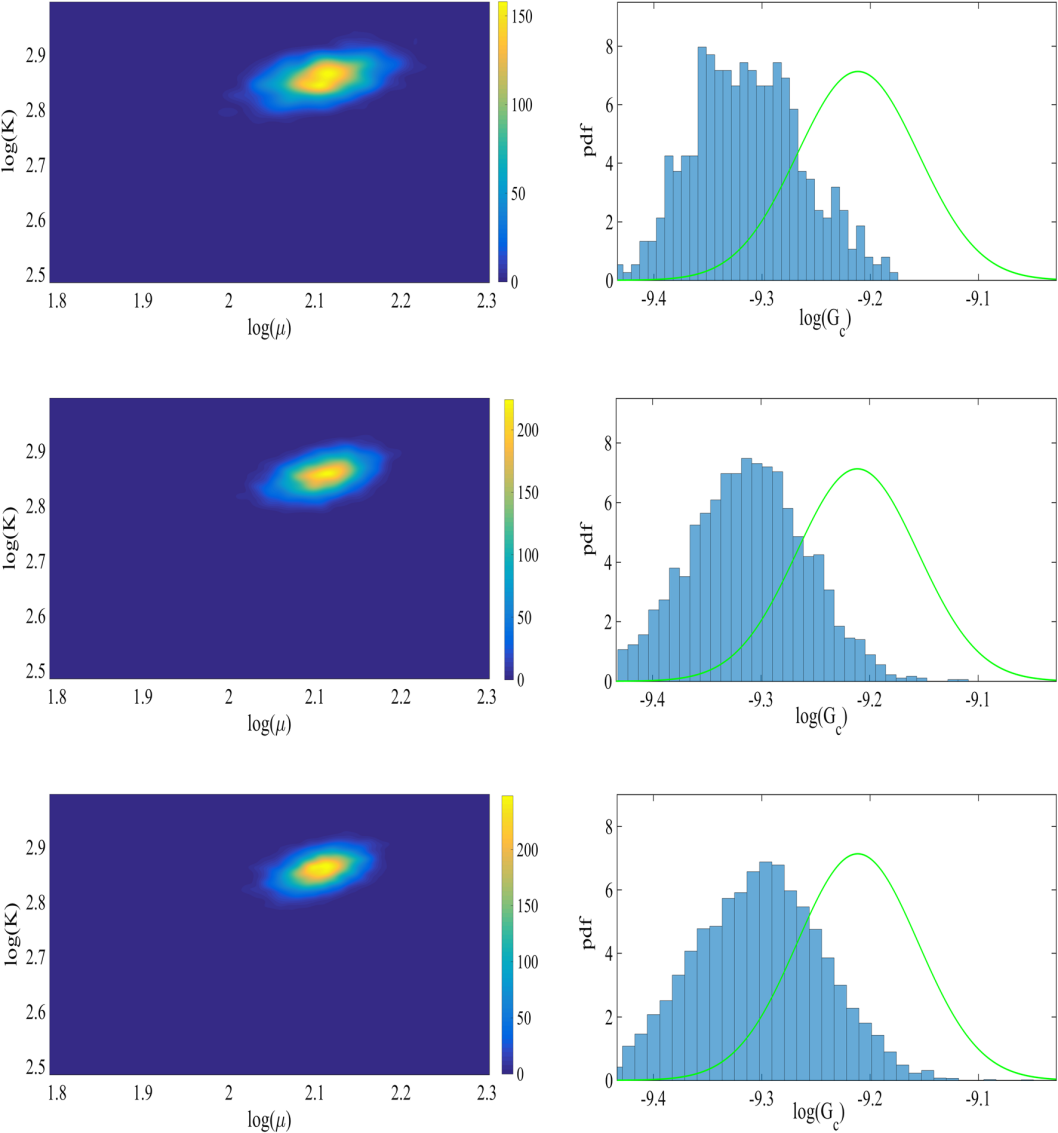
Left: the joint probability density of the elasticity parameters. Right: the prior (green line) and the posterior (histogram) of $G_c$ for the DENT example (Example 2). For the posterior distribution, we used $3\,000$ samples (the first row), $15\,000$ samples (the second row), and $50\,000$ samples (the third row)

As noted above, the phase-field solution depends on *h* and $\ell$. A detailed computational analysis was for instance performed in [5, 6]. In general, for smaller *h* (and also smaller $\ell$) the crack path is better resolved, but leads to a much higher computational cost.

Figure 10 illustrates the crack pattern using different mesh sizes varying between $h=1/20$ and $h=1/320$. For these mesh sizes, we show the load-displacement diagram in Fig. 11 and the corresponding CPU time in Table 3.

Here we strive to solve the problem using a coarse mesh and employ MCMC to find parameters that make the solution more precise compared with the reference value. Figure 12 shows the obtained displacement with both prior and posterior distribution for $h=1/20$. The efficiency of the Bayesian estimation is pointed out here since the peak point and the failure point are estimated precisely. The posterior distributions are shown in the right panel as well. The estimation can also be performed for finer meshes: Figs. 13 and 14 illustrate the load-displacement curves for $h=1/40$ and $h=1/80$, respectively. In both cases, in addition to the precise estimation of the crack-initiation point and the material-failure point, the curve is closer to the reference value. Again, the posterior distributions are shown on the right panels. Finally, the mean values of the posterior distributions in addition to their acceptance rates are indicated in Table 2.

### Example 2: Double edge notch tension (DENT) test

This numerical example is a fracture process that occurs through the coalescence and merging of two cracks in the domain. We consider the tension test with a double notch located on the left and right edge. The specimen is fixed on the bottom. We have traction-free conditions on both sides. A non-homogeneous Dirichlet condition is applied to the top-edge. The domain has a predefined two-notch located in the left and right edge in the body as shown in Fig. 15a. We set $A:=20\,\mathrm {mm}$ and $B:=10\,\mathrm {mm}$ hence $\Omega =(20,10)^2\,\mathrm {mm^2}$. For the double-edge-notches, let $H_1:=5.5\,\mathrm {mm}$ and $H_2:=3.5\,\mathrm {mm}$ with the predefined crack length of $l_0:=5\,\mathrm {mm}$ (Fig. 15a). This numerical example is computed by imposing a monotonic displacement ${\bar{u}}=1\times 10^{-4}$ at the top surface of the specimen in a vertical direction. The finite element discretization that uses $h=1/80$ is indicated in Fig. 15b.

According to the truncated KL-expansion, for the bulk modulus *K*, Eq. 37 gives the mean value of ${\bar{K}}=23.58$ and the standard deviation of $\sigma _{K}=0.28$. Therefore, the parameter varies between $10\,\mathrm {kN/mm^2}$ and $14\,\mathrm {kN/mm^2}$. For the shear modulus, the expectation of $\bar{\mu }=22.8$ and the standard deviation of $\sigma _\mu =0.23$ leads to the variation range $(6\,\mathrm {kN/mm^2},10\,\mathrm {kN/mm^2})$. Similarly, by using a KL-expansion for $G_c$, we obtained the variation range between $8\times 10^{-5}\,\mathrm {kN/mm^2}$ and $12\times 10^{-5}\,\mathrm {kN/mm^2}$. Figure 16 illustrates the effect of their different values including shear and bulk modulus and $G_c$ on the curve.

We assumed the uniform proposal distribution, namely the normal distribution

44 $$\begin{aligned} \mathcal {K}(\theta \rightarrow \theta ^*):=\frac{1}{\sqrt{2\pi \sigma ^2}}\exp \left( -\frac{(\theta -\theta ^*)^2}{2\sigma ^2} \right) . \end{aligned}$$

As we aforementioned, the random field can be represented using the KL-expansion. In this example (32) is employed to parameterize the elasticity and energy rate parameters. The random perturbations are imposed on the $\xi$ coefficients in the KL expansion. According to the proposal, the mean of the KL-expansion is updated.

Here we plan to study the effect of the number of samples *N* on the posterior distribution. Figure 17 shows the joint distributions of (${K}$, $\mu$), and the marginal distribution of $G_c$ using $N=3\,000$, $N=12\,000$, and $N=50\,000$. The calculations are done with $h=1/80$, and $h=1/320$ is used as the reference. As shown, with a larger number of samples, the distribution is close to a normal distribution. Table 4 points out the mean values in addition to the acceptance rate of all influential parameters.

**Table 4:** The mean value and the acceptance rate of the posterior distributions of $\mu$, *K*, and $G_c$ with $N_\text {samples}=3\,000$, $N_\text {samples}=12\,000$, and $N_\text {samples}=50\,000$ in the DENT example (Example 2)

	$N_\text {samples}$=3 000		$N_\text {samples}$=15 000		$N_\text {samples}$=50 000	
	Mean ($\mathrm {kN/mm^2}$)	Rate (%)	Mean ($\mathrm {kN/mm^2}$)	Rate (%)	Mean ($\mathrm {kN/mm^2}$)	Rate (%)
$\mu$	8.26	31.0	8.23	31.5	8.35	36.2
${K}$	17.4	31.0	17.42	31.5	17.12	36.2
$G_c$	$9.02\times 10^{-5}$	15.2	$9.1\times 10^{-5}$	15.8	$9.68\times 10^{-5}$	16.7

All units are in $\mathrm {kN/mm^2}$

**Fig. 18:**
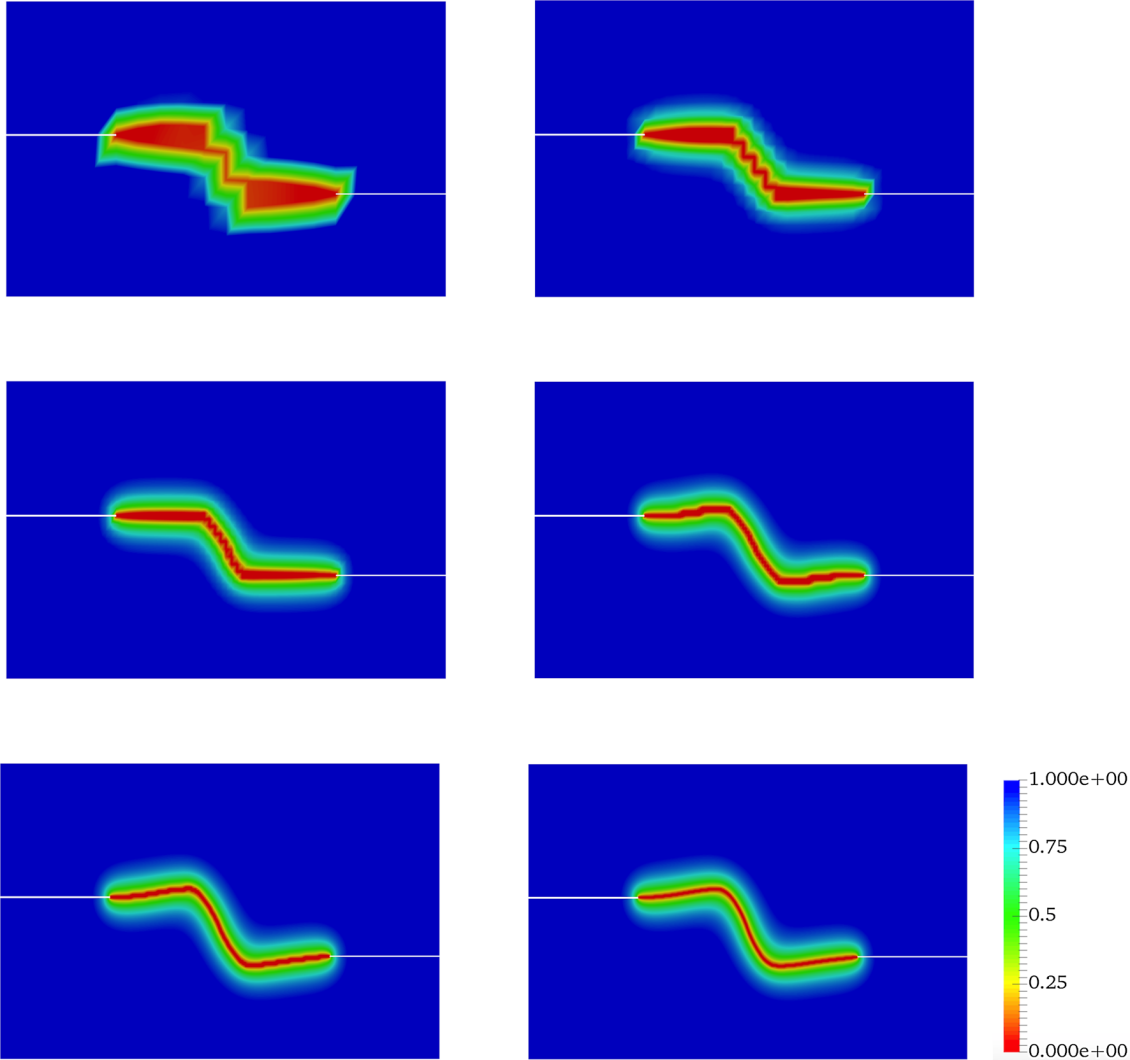
The effect of the mesh size on the crack propagation in the DENT example (Example 2). The mesh sizes are (from the left) $h=1/10$, $h=1/20$, $h=1/40$, $h=1/80$, $h=1/160$, and $h=1/320$ (the reference)

**Fig. 19:**
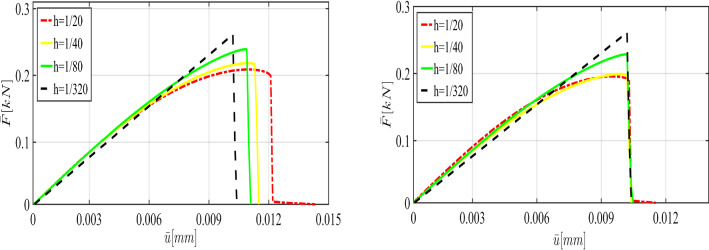
The load-displacement curve of DENT (Example 2) with different mesh sizes. Here the parameters are chosen according to the prior (left) and posterior (right) distributions

As the next step, we use different mesh sizes for the Bayesian inversion using 15 000 samples. Figure 18 shows the crack pattern using different mesh sizes changing from $h=1/10$ to $h=1/320$. Finer meshes lead to a smoother and more reliable pattern. Figure 19 depicts the load-displacement diagram using the prior values. With coarse meshes, the curve is significantly different from the reference including crack initiation. Using Bayesian inversion (see Fig. 17) enables us to predict the crack propagation and initiation more precisely. As the figure shows, even for the coarsest mesh (compare $h=1/10$ to $h=1/320$) the peak and fracture points are estimated precisely. For finer meshes (e.g., $h=1/80$) the diagram is adjusted tangibly compared to the reference value. Finally, a summary of the mean values (of posterior distributions) and their respective acceptance rate is given in Table 5.

The significant advantage of the developed Bayesian inversion is a significant computational cost reduction. As shown, for SENT and DENT, by using Bayesian inference for coarser meshes, the estimated load-displacement curve is very close to the reference values. We should note that the needed CPU time for $h=1/320$ is approximately 4.5 hours; however the solution with $h=1/80$ is obtained in less than 10 minutes. This fact pronounces the computational efficiency provided by Bayesian inversion, i.e., obtaining a relatively precise solution in spite of using much coarser meshes.

**Table 5:** The mean of the posterior distributions of $\mu$, *K*, and $G_c$ for different mesh sizes in the DENT example

	$\mu$	Rate (%)	${K}$	Rate (%)	$G_c$	Rate (%)
$h=1/20$	8.90	33.3	18.08	33.3	$8.15\times 10^{-5}$	13
$h=1/40$	8.15	37.5	17.61	37.5	$8.23\times 10^{-5}$	15.2
$h=1/80$	8.35	38	17.4	38	$9.10\times 10^{-5}$	26.4

The units are in $\mathrm {kN/mm^2}$

**Fig. 20:**
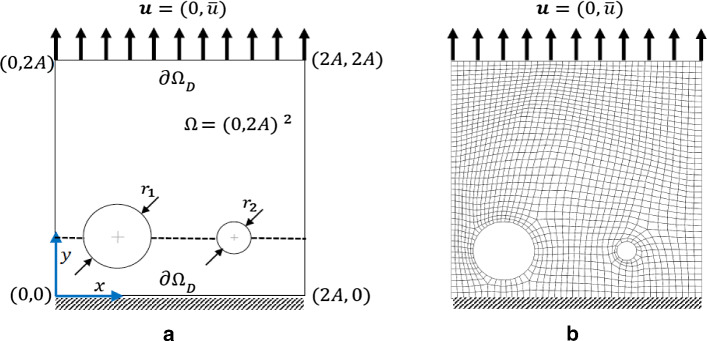
Schematic of SENT with voids (Example 3) (left) and its corresponding meshes with $h=1/40$ (right)

### Example 3: Tension test with two voids

Here we consider the tension test where two voids are located in the domain as a more complicated example. The voids are used to weaken the material and to lead to crack nucleation/initiation without an initial singularity (i.e., a pre-existing crack). The specimen is fixed on the bottom. We have traction-free conditions on both sides. A non-homogeneous Dirichlet condition is applied to the top. Domain includes a predefined two voids in the body, as depicted in Fig. 20a. We set $$A=0.5\,\mathrm {mm}$$ hence $$\Omega =(0,1)^2\,\mathrm {mm^2}$$. The radius of left void is $$r_1:=0.247 $$ with the center $$c_1:=(0.21,0.197)$$. The radius of the right void is $$r_2:=0.0806$$ with the center $$c_2:=(0.7,0.197)$$. This numerical example is computed by imposing a monotonic displacement $${\bar{u}}:=1\times 10^{-4}$$ at the top surface of the specimen in vertical direction. The finite-element discretization corresponding $$h=1/40$$ is shown in Fig. 20b.

**Fig. 21:**
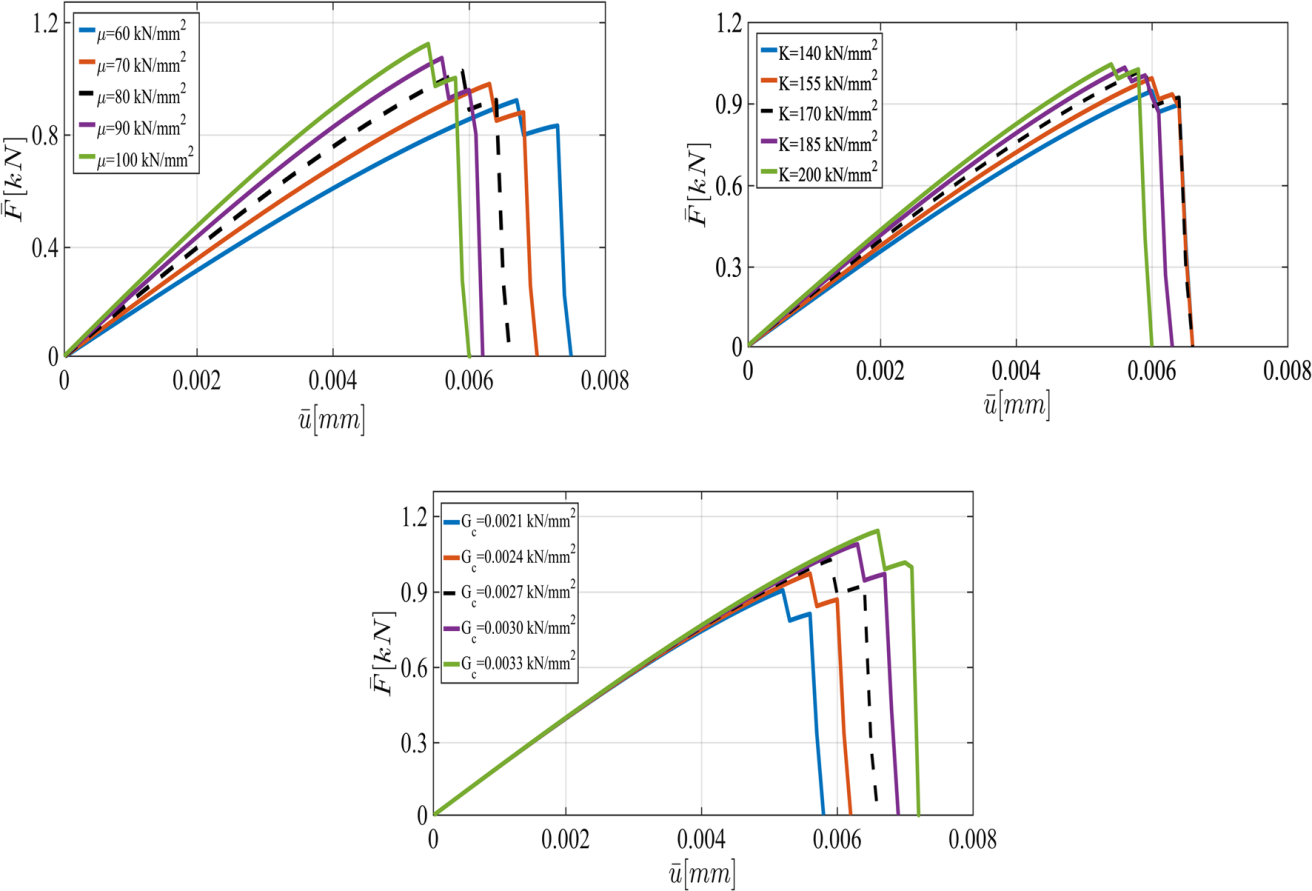
The load-displacement curve for different values of $\mu$ (top left), *K* (top right) and $G_c$ (bottom) for the SENT voids example (Example 3)

**Fig. 22:**
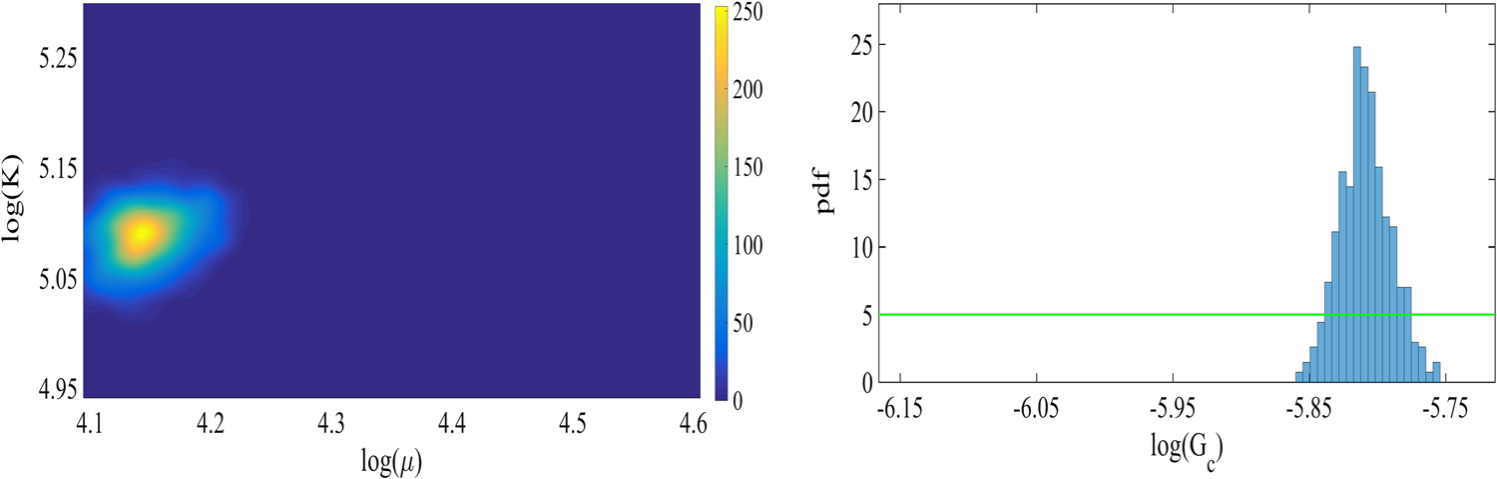
Left: the joint probability density of the elasticity parameters. Right: the prior (green line), and the posterior (histogram) distribution of $G_c$ for SENT with voids (Example 3)

Due to the resemblance to the first example (SENT) we use the same range of parameters and the variables are again spatially constant random variables. The load-displacement curves obtained from different values of $\mu$, ${K}$, and $G_c$ are illustrated in Fig. 21.

This numerical example includes two voids results in multi-stage crack propagation. Hence, in the load-displacement curve, two peak points exist to demonstrate multi-stage crack propagation, see Fig. 21.

Figure 22 shows the proposal distribution where a uniform prior distribution is used for Bayesian inversion with 10 000 samples. Here we use $h=1/160$ as the reference solution and $h=1/80$ is employed to estimate the parameters. In summary, the mean values are $\mu =63\,\mathrm {KN/mm^2}$, $K=162\,\mathrm {KN/mm^2}$, and $G_c=0.003\,\mathrm {KN/mm^2}$, and the acceptance rates are 28% (the elasticity parameters) and 21% (the critical energy rate).

**Fig. 23:**
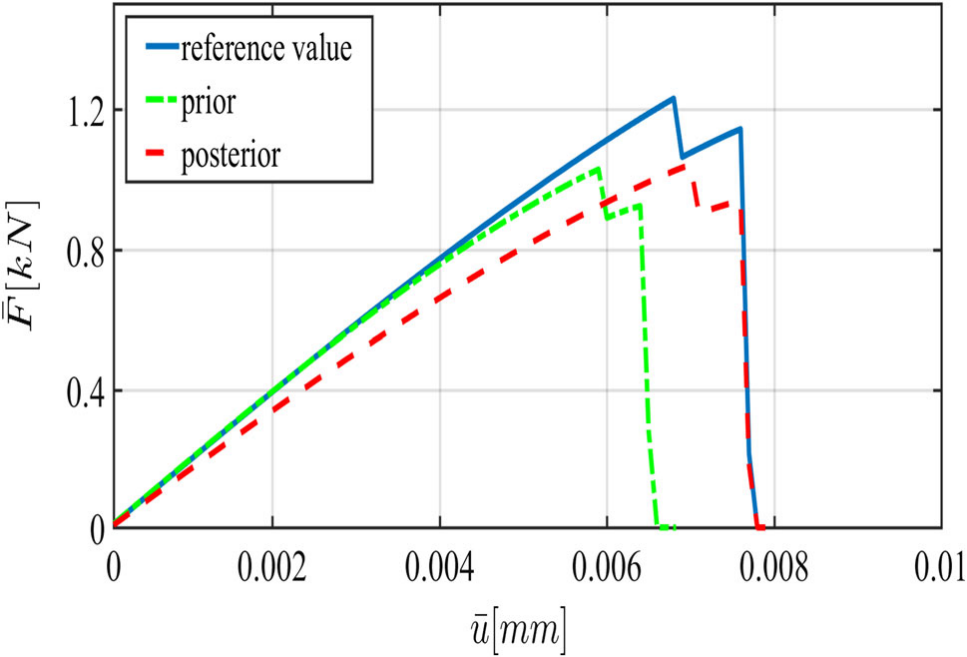
The load-displacement diagram of SENT with voids (Example 3). The parameters are the mean values ($\mu =80\,\mathrm {kN/mm^2}$, $K=170\,\mathrm {kN/mm^2}$, and $G_c=2.7\times 10^{-3}\,\mathrm {kN/mm^2}$) obtained by the three-dimensional Bayesian inference

We solve the forward model with the mean values of the estimated parameters. As Fig. 23 shows, the difference between the prior distribution and the reference solution is significantly large. By using Bayesian inversion, we could compensate this difference; crack initiation and material failure points are estimated precisely. Although multidimensional Bayesian inversion increases the computational costs (CPU time), the estimated solution is closer to the reference value.

**Fig. 24:**
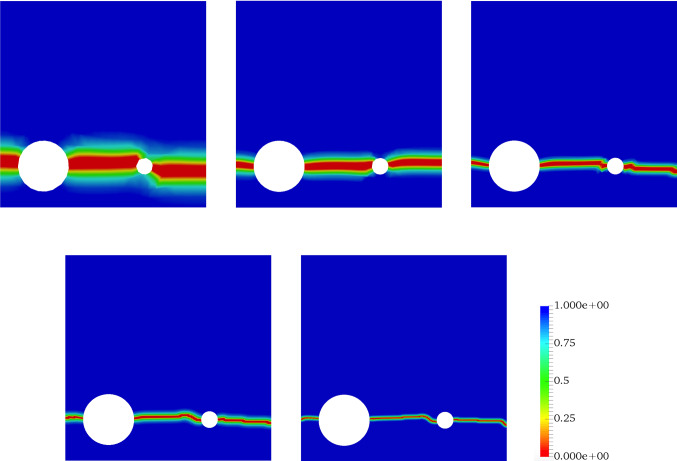
The effect of the mesh size on the crack propagation. The mesh sizes are (from the left) $h=1/10$, $h=1/20$, $h=1/40$, $h=1/80$, and $h=1/160$ (the reference)

**Fig. 25:**
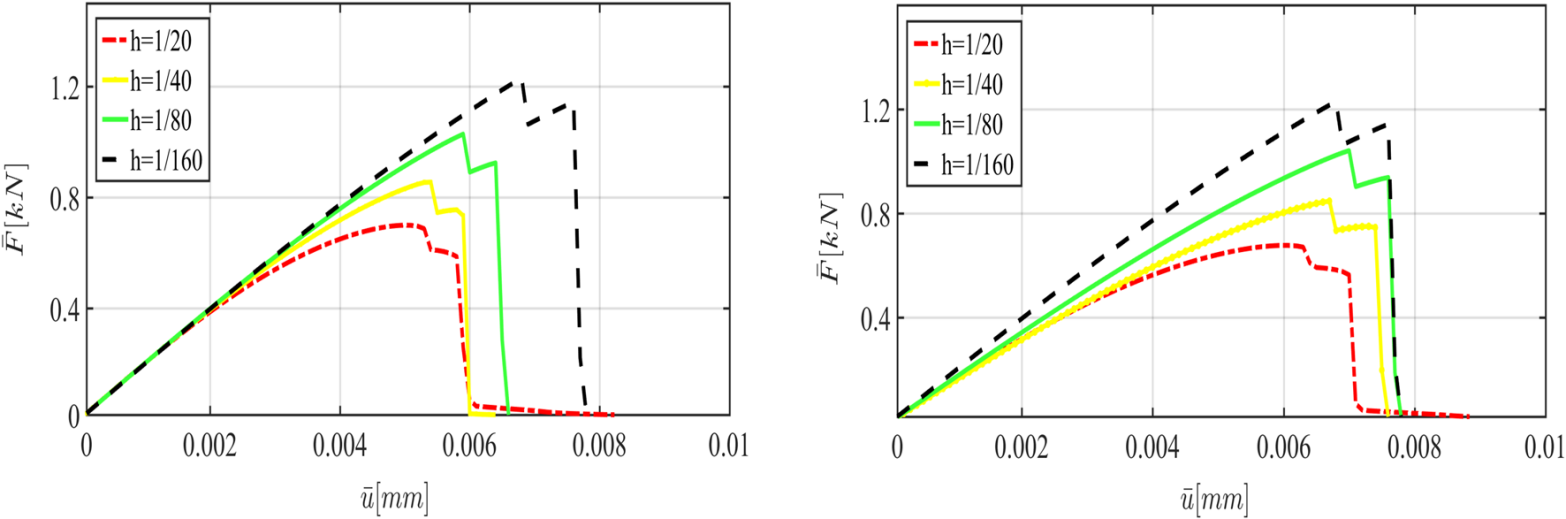
The load-displacement curve for different mesh sizes for the tension test with voids. The effective parameters are chosen according to the prior (left) and posterior (right) distributions

Finally, we show the crack patterns obtained by different meshes varying from $h=1/10$ to $h=1/160$. We use Bayesian inference to estimate the unknown parameters with the three-dimensional approach for $h=1/20$ and $h=1/40$. As Fig. 25 illustrates, although the solution based on the posterior distribution is more precise (i.e., a better estimation of crack initiation and the fracture point) compared to the one based on the prior distribution, there is still a difference compared to the reference value. These results conform to Fig. 24, since the estimated crack pattern is considerably larger than the reality.

## Conclusions and future works

In this work, we proposed a Bayesian approach to estimate material parameters for propagating fractures in elastic solids. For the fracture model, we adopted a phase-field approach. For the parameter estimation, we employed a Bayesian framework. We studied three phase-field fracture settings, and in each one, bulk and shear modulus as well as the critical elastic energy release rate were estimated with respect to a reference solution.

The developed Bayesian framework enabled us to provide useful knowledge about unknown parameters. By using Bayesian inversion, we could estimate the load-displacement curve precisely even with coarse meshes. For instance, in the first example (SENT), the diagram for $h=1/320$ and $h=1/80$ are essentially same, although a noticeable CPU time reduction is achieved. Interestingly, using even coarser meshes, the crack initiations and material fracture times can be estimated very well in all examples.

As one future application, the Bayesian approach will be used in multiscale problems to study crack propagation in heterogeneous materials, e.g., in composites. Due to their complexities, Bayesian inference will be employed to estimate material properties when the fiber-reinforced structures have a random distribution.
